# FOXM1 binds directly to non-consensus sequences in the human genome

**DOI:** 10.1186/s13059-015-0696-z

**Published:** 2015-06-23

**Authors:** Deborah A. Sanders, Michael V. Gormally, Giovanni Marsico, Dario Beraldi, David Tannahill, Shankar Balasubramanian

**Affiliations:** Cancer Research UK, Cambridge Research Institute, Li Ka Shing Center, Robinson Way, Cambridge, CB2 0RE UK; Department of Chemistry, University of Cambridge, Lensfield Road, Cambridge, CB2 1EW UK; School of Clinical Medicine, The University of Cambridge, Addenbrooke’s Hospital, Hills Road, Cambridge, CB2 0SP UK; Present address: Domainex, 162 Cambridge Science Park, Milton Road, Cambridge, CB4 0GH UK

## Abstract

**Background:**

The Forkhead (FKH) transcription factor FOXM1 is a key regulator of the cell cycle and is overexpressed in most types of cancer. FOXM1, similar to other FKH factors, binds to a canonical FKH motif *in vitro*. However, genome-wide mapping studies in different cell lines have shown a lack of enrichment of the FKH motif, suggesting an alternative mode of chromatin recruitment. We have investigated the role of direct versus indirect DNA binding in FOXM1 recruitment by performing ChIP-seq with wild-type and DNA binding deficient FOXM1.

**Results:**

An *in vitro* fluorescence polarization assay identified point mutations in the DNA binding domain of FOXM1 that inhibit binding to a FKH consensus sequence. Cell lines expressing either wild-type or DNA binding deficient GFP-tagged FOXM1 were used for genome-wide mapping studies comparing the distribution of the DNA binding deficient protein to the wild-type. This shows that interaction of the FOXM1 DNA binding domain with target DNA is essential for recruitment. Moreover, analysis of the protein interactome of wild-type versus DNA binding deficient FOXM1 shows that the reduced recruitment is not due to inhibition of protein-protein interactions.

**Conclusions:**

A functional DNA binding domain is essential for FOXM1 chromatin recruitment. Even in FOXM1 mutants with almost complete loss of binding, the protein-protein interactions and pattern of phosphorylation are largely unaffected. These results strongly support a model whereby FOXM1 is specifically recruited to chromatin through co-factor interactions by binding directly to non-canonical DNA sequences.

**Electronic supplementary material:**

The online version of this article (doi:10.1186/s13059-015-0696-z) contains supplementary material, which is available to authorized users.

## Background

FOXM1 is a member of the Forkhead family of transcription factors and is a master regulator of the cell cycle [1–3]. There is growing evidence that FOXM1 is also involved in the regulation of many other cellular processes through the activation of specific transcriptional pathways. FOXM1 is frequently overexpressed in cancer [4–6] and is linked with many processes involved in oncogenesis, such as metastasis [7, 8], cancer stem cell proliferation [9, 10], and angiogenesis [11, 12]. Dysregulated FOXM1 expression is an early initiating event in cancer [13, 14] and as such it represents a novel therapeutic target.

FOXM1-regulated processes are mediated through the transactivation of key genes by FOXM1 protein binding to target sequences in gene promoters [2]. In common with other members of the Forkhead family, FOXM1 contains a highly conserved DNA binding domain (DBD) [15], which *in vitro* binds to DNA sequences containing a canonical FKH motif (RYAAAYA) [16, 17]. In humans there are over 40 different Forkhead family members with diverse biological functions [18] and it is currently unclear how different Forkhead factors are recruited to specific genomic sites to regulate distinctly different transcriptional responses.

A number of genome-wide studies have mapped FOXM1 binding to the FKH target motif, while others have mapped the indirect binding of FOXM1 through its interaction with B-Myb or LIN9, a component of the MuvB complex [19]. These studies present conflicting models of FOXM1 recruitment to chromatin binding sites. For example, Sadasivam *et al.* [19] using HeLa cells, found that the FKH motif was enriched in genomic sites bound by LIN9 and B-Myb, which were predominantly located within cell cycle promoters. These data suggest that FOXM1 directly binds to the FKH consensus and is co-bound by the MuvB complex and B-Myb. In contrast, Chen *et al.* [20] found no enrichment of the FKH consensus at FOXM1 binding sites in U2OS cells. This latter study identified just 270 sites total, again located primarily in promoter regions associated with cell cycle genes. The majority of these sites overlapped with the LIN9/B-Myb binding sites uncovered by Sadasivam *et al.* [19]. This study suggested an alternative mechanism of FOXM1 recruitment to chromatin whereby FOXM1 protein directly interacts with the MuvB/B-Myb complex rather than at FKH sequences. Our previous ChIP-seq analysis of FOXM1 binding [21] in two breast cancer cell lines appears to support existence of both modes of recruitment. For both MCF7 and MDA-MB-231 cells, while only 35 and 15 % of peaks respectively contained the FKH consensus motif these were significantly enriched over background.

Conventional recruitment of transcription factors to genomic binding sites is based on a model of direct binding at high affinity consensus motif sequences in *cis*-regulatory regions. However, there are many examples of non-canonical modes of transcription factor binding, including tethering, where a transcription factor is recruited by other protein complexes previously assembled at the DNA target site. For example, genome-wide binding studies of ER**α**, have identified sites lacking an ERE (estrogen response element), in which binding is mediated by tethering to a large repertoire of pre-assembled DNA-binding transcription factors including AP-1 [22]. Another common mechanism that may be used together or independently of tethering, is the recognition by a transcription factor of lower affinity non-consensus binding sites.

Additionally, some transcription factors show different modes of recruitment to chromatin at specific sub-sets of genomic binding sites. For example, the ETS family member ELK1 [23] has two distinct types of binding modes, either binding redundantly with other ETS factors at regulatory sites or uniquely to different binding sites leading to the regulation of different transcriptional programs. Similarly, ER**α** was recently shown to have two modes of binding, one present in shared sites across multiple cell lines with high-affinity ERE sequences and the other cell line-specific that is defined by the lack of ERE consensus motifs and co-occurrence of a distinct set of transcription factors [24]. A similar mechanism may also apply to FOXM1 recruitment, thereby explaining the presence of sites without a consensus FKH binding site. Such alternative mechanisms of transcription factor recruitment are thought to increase regulatory flexibility through the recognition of a wider repertoire of sites mediated by combinations of different sets of co-factors [22].

Deciphering the precise mechanism of FOXM1 recruitment to genomic binding sites is of key importance to understand how *in vivo* binding specificity is achieved. This has become of great interest due to our recent proof-of-principle for the potential therapeutic of targeting the DBD of FOXM1 by small molecules to prevent chromatin recruitment and transactivation [25, 26], thus it is important to elucidate which binding sites represent direct binding versus indirect events. The aim of this study was to elucidate the details of FOXM1 binding genome-wide, by exploring the role of direct versus indirect FOXM1 recruitment and the mechanism of binding at sites lacking a FKH consensus sequence. Additionally, we have examined whether unique FOXM1 binding modes are characterized by any distinctive affinity binding motifs or the presence of specific protein co-factors.

## Results

### The FOXM1 DBD is essential for DNA binding *in vitro*

**Fig. 1:**
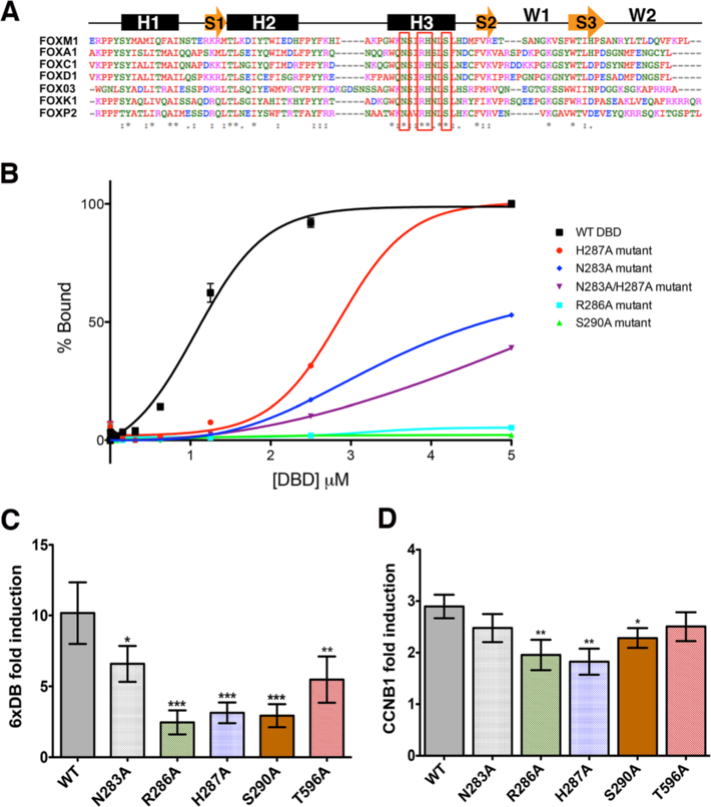
Mutation of the FOXM1 DBD inhibits DNA binding. **a** Sequence alignments of the DBD for a number of Forkhead family members with the secondary structure shown schematically above. The residues used to generate point mutations are outlined in red. (*) conserved amino acids. H1-3 are α-helices, the orange arrows are β strands, and W1-2 are winged domains. **b** Plot showing relative change of polarization of a fluorescently-labeled (6FAM) dsDNA FKH consensus oligonucleotide upon addition of increasing concentrations of GST-FOXM1 WT or mutant DBD proteins. The FP assay provides a quantitative method and non-disruptive method to determine FOXM1 affinity for target by measuring the fluorescence polarization signals from the FAM-labeled FKH consensus (see Materials and methods). Data are plotted as % binding and show mean ± SD of triplicate experiments. (WT K_d_ = 1.10 ± 0.02 μM, H287A K_d_ = 3.04 ± 0.10 μM). **c** Plots showing relative luciferase activity of a 6X DB-TATA-luciferase reporter in cells transiently transfected with either WT or DBD mutant FOXM1 with the T596A mutation as a positive control. Data are shown as fold induction of luciferase activity following doxycycline induction. **d** Plot showing fold induction of a luciferase reporter containing a 200 bp sequence taken from the CCNB1 promoter following doxycycline induction of WT and mutant FOXM1 expression. Data represent triplicate experiments ± SD. (*) *P* <0.05, (**) *P* <0.01, (***) *P* <0.001

The Forkhead DBD [27] adopts a structure consisting of three α helices, three β sheets, and two wings with the main contact points with the DNA major groove located in helix H3. Amino acid residues involved in the base-specific contacts are highly conserved among all Forkhead members [28]. To investigate the importance of direct interaction of the FOXM1 DBD with the FKH consensus on *in vitro* binding, four highly conserved H3 amino acids were chosen to generate mutations that are predicted to interfere with DNA binding. (Fig. 1a; H3 residues selected for mutation are indicated with red box). Four point mutations, N283A, R286A, H287A, S290A. and one double mutant N283A/H287A (Fig. 1a) were engineered and used to generate FOXM1 DBD-GST-tagged proteins.

The DNA binding activity of the wild-type (WT) and mutated FOXM1 proteins was assessed by fluorescence polarization (FP) binding assays using a carboxyfluorescein (6FAM) -tagged dsDNA oligonucleotide containing the FKH consensus sequence (5′AAACAAACAAACAATC). Figure 1b shows that all mutations significantly decrease the binding affinity compared to the WT FOXM1 protein. The *K*_d_ for WT FOXM1 for the consensus DNA is 1.10 ± 0.02 μM, compared to 3.04 ± 0.10 μM for H287A. For both the N283A and double mutant (N283A/H287A) binding did not reach saturation (*K*_d_ >5 μM). No significant binding was observed for the R286A or S290A mutants. While double mutation (N283A/H287A) appeared to show an additive inhibitory effect compared to either individual mutations, it still was not as effective as R286A and S290A, which led to complete loss of DNA binding.

The effect of the mutations in the FOXM1 DBD on transcriptional activity was next tested using a cell-based reporter system. Identical mutations were therefore generated in the DBD of the full-length FOXM1B protein, as this isoform is the predominantly overexpressed isoform in cancer [4, 29]. Transient transfections were performed using inducible FOXM1B constructs and the level of transactivation from the FOXM1 DBD mutations compared to WT, following addition of doxycycline. An additional control was performed based on a T596A mutation in the transactivation domain of FOXM1 that has been previously shown to reduce transcriptional activity [17]. Constructs were co-transfected into HeLa TRex cells with a firefly luciferase (FLuc) reporter (6xDB) plasmid containing six copies of the FKH consensus sequence (AAACAAACAAAC) and a renilla luciferase reporter (RLuc) plasmid to control for transfection efficiency. Figure 1c shows that all mutated FOXM1 proteins had a significant reduction in transcriptional activity compared to the WT FOXM1B construct (approximately 25-75 % lower), which had approximately 10-fold activation on induction with doxycycline.

To confirm that the FOXM1 DBD mutations reduce transcriptional activity in the context of endogenous genomic binding sites rather than the 6x FKH consensus alone, the cell reporter assay was repeated using promoter regions (approximately 200 bp) from known FOXM1 target genes, *CCNB1* and *PLK1* [30, 31], neither of which contain a FKH consensus motif. Following doxycycline treatment (1 μg/mL), the level of induction by the FOXM1 WT construct was approximately three-fold lower for expression driven by both the *CCNB1* and *PLK1* reporters than the 6x FKH consensus (Fig. 1d and Additional file 1: Figure S1). Results for both of these promoter constructs showed significantly reduced transcriptional activation following doxycycline induction with three of the FOXM1 DBD mutations compared to the WT (R286A, H267A, and S290A). The degree of reduction was lower compared to the artificial 6xDB, however the activity of these promoters is not exclusively regulated by FOXM1, for example *CCNB1* expression is known to be regulated by an NF-Y binding site contained within this 200 bp region [32] and indeed both of these natural promoter regions have a higher level of basal activity than the artificial FKH domain leading to lower levels of induction with doxycycline. In contrast, there was no significant difference observed when the assay was performed using the non-FOXM1 responsive promoters, CYP1B1 and SV40 (Additional file 1: Figure S1). These results therefore confirm that specific point mutations in the DBD can reduce the transcriptional activity of FOXM1 at an endogenous binding site.

### Generation of epitope tagged FOXM1 cell lines for ChIP-seq

To compare the genome-wide distribution of binding sites for WT and DBD mutants of FOXM1, we generated stable cell lines expressing inducible FOXM1B (WT or DBD mutants) for use in ChIP-seq experiments. The FOXM1B construct was tagged with an N-terminus GFP tag to enable ChIP-seq using an anti-GFP antibody to distinguish the expressed protein from the endogenous FOXM1. Transient transfection of FOXM1B or a GFP only control in HeLa TRex cells confirmed efficient induction of protein expression following doxycycline treatment (1 μg/mL) (Additional file 1: Figure S2A). ChIP-qPCR was then performed on five FOXM1 target genes to assess pull-down efficiency using an anti-GFP antibody. These results showed the enrichment of GFP-FOXM1 at the target genes with no detectable binding of GFP alone (Additional file 1: Figure S2B).

**Fig. 2:**
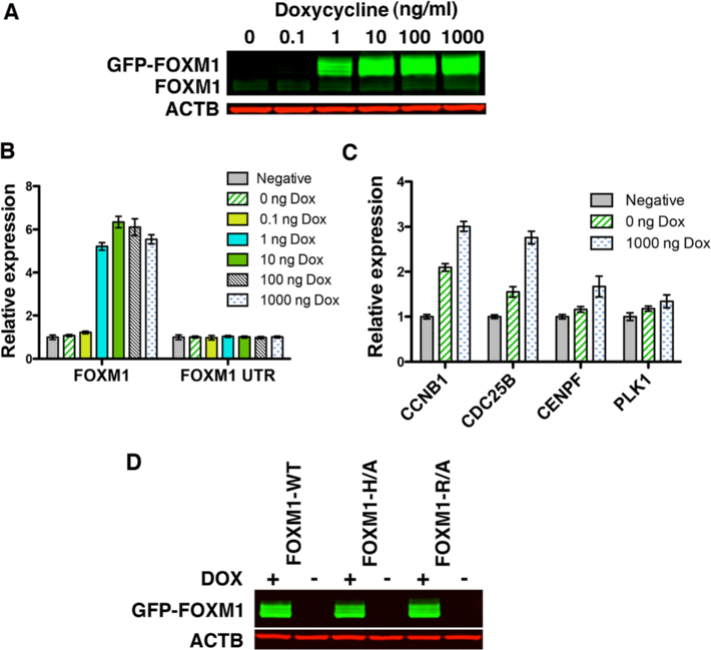
Generation of inducible GFP-FOXM1 expressing HEK293 cell lines. **a** Western blot showing induction of GFP-FOXM1B expression following addition of doxycycline for 24 h while levels of endogenous FOXM1 are unchanged. The blot was probed using antibodies for FOXM1 and ACTB. **b** Quantitative PCR (qPCR) showing RNA expression levels of total FOXM1 (GFP-FOXM1 and endogenous) and endogenous FOXM1 only (FOXM1-UTR). **c** qPCR of FOXM1 target genes transcript levels in GFP-FOXM1 cells treated ± doxycycline for 24 h and expressed relative to the levels in the parent HEK293 cells (Negative) for each transcript. Data show triplicate experiments ± SEM. **d** Western blot showing protein levels of GFP-FOXM1 in WT and mutant cell lines treated ± doxycycline (Dox) at 1 μg/mL for 24 h

The Flp-In system (Invitrogen) was next used to generate stable cell lines expressing GFP-FOXM1 WT by the targeted insertion of an expression construct at a single transcriptionally active genomic site. This system was utilized to ensure that the WT and mutant FOXM1 proteins were expressed at equivalent levels following induction. HEK293Flp-In cells stably expressing the Tet repressor at high levels were first generated by transfection of a TetR plasmid under the control of a CMV promoter. These cells were transfected with WT or mutant FOXM1-GFP plasmids together with Flp recombinase plasmid to generate inducible cell lines. In the absence of doxycycline, no detectable expression of the GFP-FOXM1 protein or transcript was observed (Fig. 2a and b), while expression was induced after addition of doxycycline at concentrations above 1 ng/mL, giving approximately 50-fold higher levels of total FOXM1 protein (1000 ng/mL) compared to the uninduced cells. There was no significant change in the level of the endogenous FOXM1 protein or mRNA following overexpression of the GFP-FOXM1 as shown by western blotting and qPCR for the FOXM1 UTR [33]. This result contrasts the hypothesis, proposed by Halasi *et al*. [33], that FOXM1 expression is primarily regulated by a positive auto-regulatory loop. Isoform specific qPCR (Additional file 1: Figure S3) showed that only GFP-FOXM1B protein was significantly upregulated following doxycycline induction.

Doxycycline induction of WT GFP-FOXM1 led to a small but significant increase in transcript levels for a number of known FOXM1 target genes as assessed by qPCR (Fig. 2c). *CCNB1* and *CDC25B* levels increased by approximately 2.8 fold, while *CENPF* showed a smaller increase of approximately 1.3-fold when compared to the parental HEK293 TetR cell line (negative) and there was no significant change in levels of *PLK1*. When expression is compared before and after the addition of doxycycline all four genes showed a slight increase in expression compared to the parental cell line. The generally low level of induction observed is similar to that described in other FOXM1 overexpression studies [3, 31].

These results confirm that GFP-tagged WT FOXM1B recapitulates endogenous FOXM1 recruitment at genomic targets to elicit a transcriptional response. Next, stable cell lines were generated that express FOXM1 comprising either the R286A or H287A point mutation to allow comparison of the binding distributions of WT and DBD mutated FOXM1. These mutations were selected based on the observed changes in the FP binding data and also in the luciferase activity assays described earlier (Fig. 1). The level of DBD mutant GFP-FOXM1 was then compared to the WT following doxycycline induction (1 μg/mL) by western blotting (Fig. 2d) to confirm similar protein expression levels in all three cell lines, which is critical for a precise comparison of binding by ChIP-seq. Sequencing also confirmed that each cell lines contained the correct mutation in the GFP-FOXM1 (Additional file 1: Figure S4) and indirect immunofluorescence using an anti-GFP antibody showed nuclear localization of the WT and mutant GFP-FOXM1 proteins in the majority of cells (Additional file 1: Figure S5).

### GFP-FOXM1 binding recapitulates endogenous FOXM1 binding

To confirm that the genomic distribution of GFP-FOXM1 expressed protein matched that of endogenous FOXM1, ChIP-seq data were compared between HEK 239 Flp-In and HEK293 GFP-FOXM1 cells using with a FOXM1 or GFP antibody, respectively, following induction with doxycycline (1 μg/mL). In addition, the genomic binding of GFP in GFP-only expressing cells was tested, this was done by ChIP-qPCR as it was not possible to generate a sequencing library due to very low levels of enrichment following pull-down. No enrichment of 10 known FOXM1 binding sites was observed in the GFP-only expressing cell line compared to the GFP-FOXM1 WT cells (Additional file 1: Figure S6). This confirms that binding of GFP-FOXM1 is due to the specific interaction of FOXM1 with target DNA rather than non-specific GFP recruitment.

**Fig. 3:**
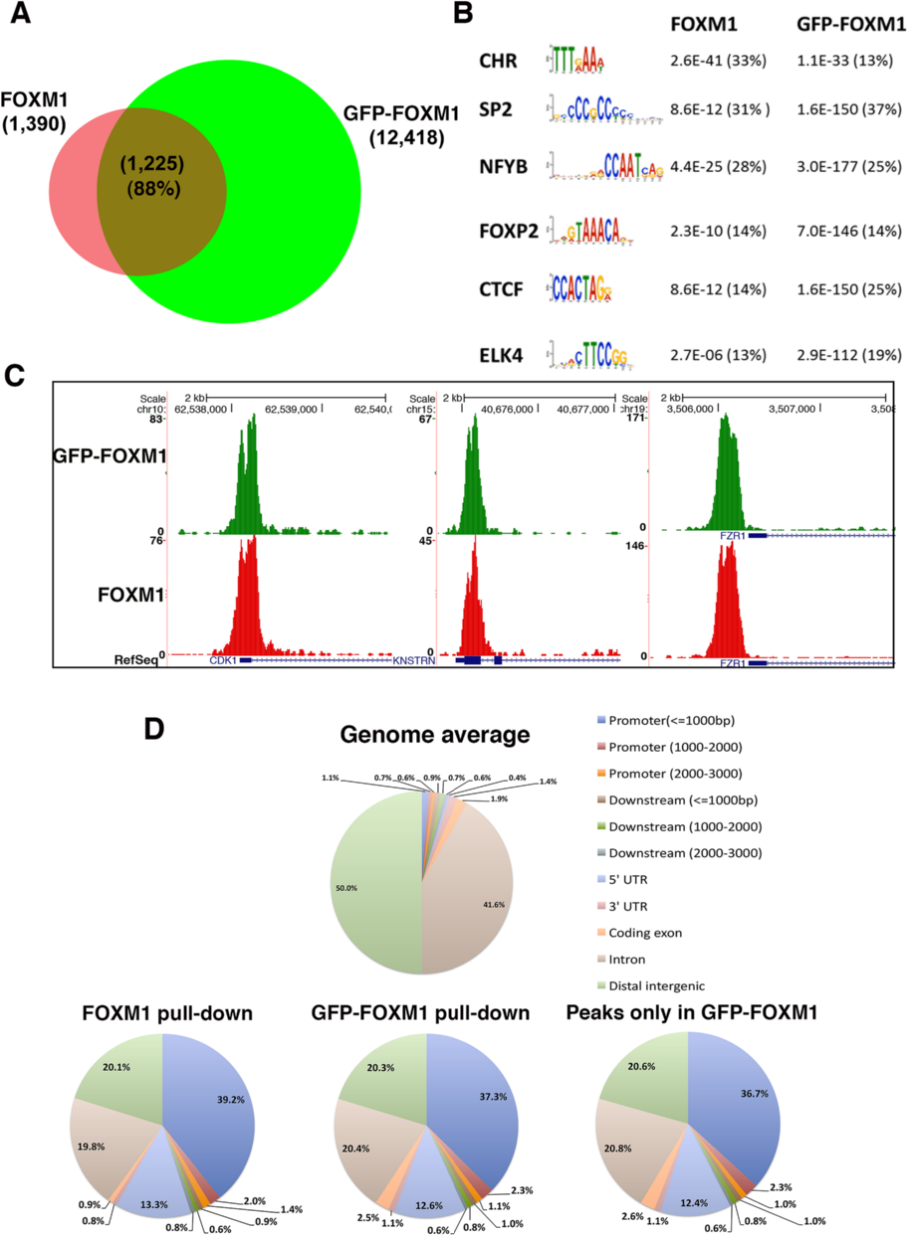
GFP-tagged FOXM1 shows a similar genomic distribution to endogenous FOXM1. **a** Venn diagram showing the overlap between endogenous FOXM1 and GFP-FOXM1 binding sites in TetR HEK293 cells, using peaks in common in two replicates for each condition. **b** Motifs enriched in FOXM1 and GFP-FOXM1. Selected motifs identified in FOXM1 are also found in the GFP-FOXM1 peaks (full list in Additional file 1: Table S2). *P* values and percentages in brackets represent the statistical significance and the percentage of peaks for each motif, respectively. Motif sequences are sorted according to their proportional representation in FOXM1 peaks. **c** Examples of genomic regions showing three representative sites in common for endogenous FOXM1 and GFP-FOXM1 binding. **d** CEAS analysis comparing genomic distribution of endogenous FOXM1 binding events to GFP-FOXM1 in HEK293 cells

Two biological replicates were performed for both cell lines and 25–30 million reads were obtained for each sample (Additional file 1: Table S1) and binding peaks were called using Model-based Analysis for Chip-Seq (MACS) [34]. For endogenous FOXM1, 1,390 peaks were present in both replicates and for GFP-FOXM1 12,418 peaks were identified (Additional file 2), with a high level of concordance between replicates (approximately 78 % for FOXM1 and approximately 81 % for GFP-FOXM1). Comparison of the endogenous FOXM1 and GFP-FOXM1 peak locations (Fig. 3a) showed a high degree of overlap (approximately 88 %) indicating that the GFP-FOXM1 protein captures the majority of the endogenous FOXM1 binding sites. In the endogenous FOXM1 replicates approximately 50 % more peaks were called in one sample (3,970 compared to 1,722). When less stringent parameters were used for peak calling, using each replicate separately, the number of binding sites shared between endogenous FOXM1 and the GFP-FOXM1 increased to 3,173 representing approximately 26 % of the total GFP-FOXM1 binding sites. The approximately 10-fold increase in high confidence peaks called in the GFP-FOXM1 samples compared to endogenous FOXM1 is possibly due to the higher affinity of the GFP antibody and the relatively poor performance of commercial FOXM1 antibodies in ChIP-seq or the increased level of GFP-FOXM1 compared to endogenous FOXM1 protein in these cell lines.

Motif analysis using MEME (Multiple Em for Motif Elicitation) [35] of the FOXM1 and GFP-FOXM1 datasets revealed similar sets of enriched sequences suggesting that the presence of the GFP tag does not significantly affect the binding specificity of FOXM1 (Additional file 1: Table S2). The six highest enriched motifs (ranked by FDR) in the endogenous FOXM1 pull-down are shown in Fig. 3b and the *P* values from the GFP-FOXM1 are shown for comparison. Both sets contained the FKH motif as well as the CHR and CCAAT (NFYA) box motifs that have also previously been shown to be associated with FOXM1 binding [19, 20]. The percentage of peaks containing the FKH motif was similar in both sets (approximately 14 %) and critically when the peaks present only in the GFP-FOXM1 dataset were analyzed, these also showed a similar significant enrichment of the FKH motif (1.73E-152) in approximately 14 % peaks (Additional file 1: Table S2). Although the FKH motif is enriched, the relative number of peaks containing the consensus sequences is lower than that reported for some other transcription factors such as POU5F1, where approximately 70 % of peaks match the POU5F1 PWM motif [36]. This therefore highlights the observation that the majority of FOXM1 peaks, both in the endogenous and epitope tagged ChIP-seq, do not contain a consensus FKH motif.

In both the FOXM1 and GFP-FOXM1 datasets, peaks were identified in the promoter regions of known FOXM1 target genes including *CDK1* [30], *FZR1* [20], and *KNSTRN* [20] (Fig. 3c), confirming that the GFP tag does not affect FOXM1 recruitment *in vivo*. Manual inspection of some of the unique GFP-FOXM1 peaks revealed small peaks at the same location in the endogenous FOXM1 dataset. This suggests that additional peaks present in the GFP-FOXM1 dataset are low affinity endogenous sites, in which the endogenous FOXM1 ChIP-seq shows insufficient enrichment over the input to be considered significant by the MACS peak caller (Additional file 1: Figure S7A). Furthermore, analysis of read counts in all the 12,418 regions identified as peaks in the GFP-FOXM1 shows high correlation (r = 0.70-0.75) with endogenous FOXM1 ChIP-seq peaks (Additional file 1: Figure S7B). This indicates that even when the endogenous FOXM1 signal is insufficient to be called as a peak, it still shows a significant correlation to the GFP-FOXM1 signal and is not present in input controls. This lends further weight to our contention that the signal detected by FOXM1-GFP ChIP-Seq is not spurious.

Comparison of the distribution of peaks across the genome using the Cis-regulatory Element Annotation System (CEAS) tool [37] also shows a very similar pattern between endogenous FOXM1 and GFP-FOXM1 (Fig. 3d), with the greatest proportion in promoter/5′ UTR regions (approximately 53 % and approximately 56 % for GFP-FOXM1 and endogenous FOXM1 respectively compared with 2.8 % for the genome average distribution). Furthermore, peaks present only in the GFP-FOXM1 and not the endogenous FOXM1 pull-down, showed a similar genomic distribution to the endogenous FOXM1, with the majority of events found in gene promoter regions. The reproducibility, distribution, and motif analysis of the additional peaks present in the GFP-FOXM1 pull-down strongly suggests that these are not non-specific and are genuine endogenous FOXM1 sites, which are beyond the level detectable with the FOXM1 antibody. A similar phenomenon has been previously observed for other transcription factors in which improved methodology using higher affinity antibodies leads to an increased number of binding sites.

Gene ontology analysis using the Reduce and Visualize Gene ontology tool (ReViGo) [38] tool of overrepresented GO terms associated with genes located within 50 kb of FOXM1 and GFP-FOXM1 binding peaks highlights an enrichment in the known functional roles for FOXM1 including cell cycle, chromosome segregation and mitotic spindle formation processes (Additional file 1: Figure S8). In addition, processes related to methylation were also identified, which is in concordance with previous studies linking FOXM1 to promoter hypermethylation [39, 40]. Analysis of enriched GO terms associated with peaks identified only in the GFP-FOXM1 dataset highlighted processes associated with S phase and M/G1 transition of the cell cycle, both of which are known functions of FOXM1 [41, 42] in addition to its major role in the G2/M transition (Additional file 1: Table S3). This functional categorization of peaks suggests that that the GFP-FOXM1 binding pattern reflects that of endogenous FOXM1.

Overall, these analyses demonstrate that the inducible GFP-FOXM1 expression system in HEK293 cells is a suitable model for FOXM1 binding studies. Recruitment occurs at biologically relevant genomic locations that are representative of endogenous FOXM1 binding, thus GFP-FOXM1 expression is able to compete with endogenous FOXM1.

### Comparison of genomic binding sites of WT GFP-FOXM1 to DBD mutants

ChIP-seq was next performed to compare the genomic binding sites of GFP-tagged WT FOXM1 to the two GFP-tagged DBD mutant versions of FOXM1. Three biological replicates were performed for the WT and H287A and two for the R286A cell line. All cells were treated for 24 h with doxycycline (1 μg/mL) prior to chromatin extraction.

**Fig. 4:**
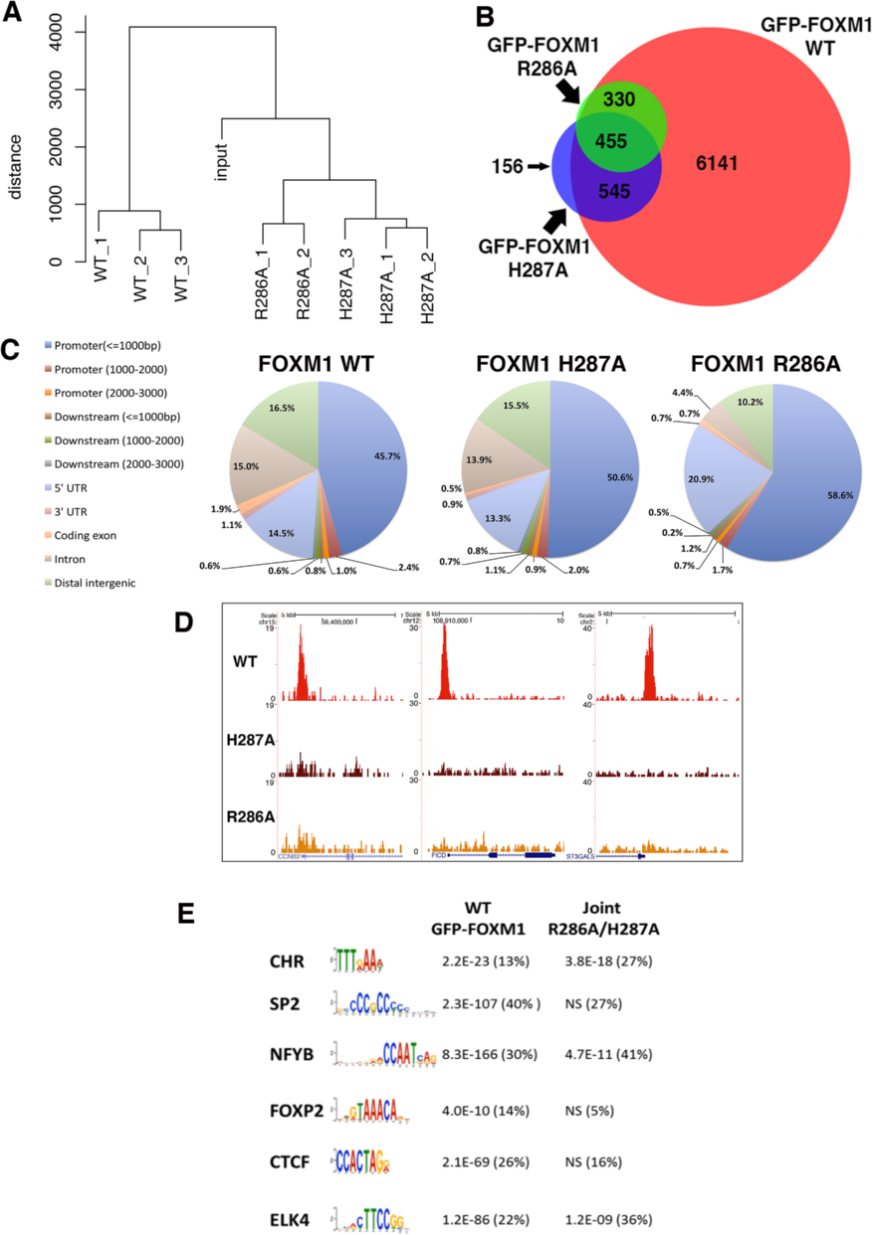
Mutation of the DBD of FOXM1 inhibits binding genome-wide. **a** Hierarchical clustering analysis of the WT and DBD mutant GFP-FOXM1 replicate ChIP-seq datasets showing that the samples separate well into distinct treatment groups. **b** Venn diagram showing the overlap between binding regions identified by ChIP-seq analysis in 293 cells expressing GFP-FOXM1 WT and the GFP-FOXM1 mutants H287A or R286A. **c** CEAS analysis comparing genomic distribution of GFP-FOXM1 binding events in WT versus DBD mutants. **d** Examples of genomic regions showing three representative promoter binding sites in which binding peaks are only identified for GFP-FOXM1 WT and are not present in the DBD mutant cell lines. **e** Motifs enriched in WT GFP-FOXM1 and the mutant R286A-H287A (full list in Additional file 1: Table S4). *P* values and percentages in brackets represent the statistical significance and the percentage of peaks for each motif, respectively. Motif sequences are sorted according to their proportional representation in WT GFP-FOXM1 peaks (NS = non-significant)

Each replicate for each cell line resulted in 25–30 million reads (Additional file 1: Table S1). For each construct, peaks in common between each replicate showed a good level of concordance. WT, H287A and R286A replicates showed 7,473 (72 % overlap), 1,169 (60 % overlap) and 804 shared binding events (81 % overlap), respectively (Additional file 2). Comparing this GFP-FOXM1 dataset with that in the preceding section again showed a high level of overlap with 6,443 shared peaks (86 % concordance), further confirming the high degree of reproducibility for the GFP-FOXM1 ChIP-seq. Furthermore, hierarchical clustering (Fig. 4a) showed that the replicate samples grouped together indicating a reproducible dataset. Overlapping the peaks from the WT and DBD FOXM1 mutants (Fig. 4b) showed that the majority of H287A (86.5 %) and R286A (98.5 %) peaks are a subset of WT GFP-FOXM1 binding sites. For H287A, although 156 peaks did not overlap with the WT or R286A peaks, most overlapped with at least one WT replicate, suggesting that they are not novel binding sites. The majority of R286A peaks are also shared by H287A (approximately 57 %). Of particular note is the observation that both FOXM1 DBD mutations significantly reduce the overall level of genomic binding. The R286A mutation was more effective at reducing binding compared to H287A, which is consistent with the *in vitro* DBD FP analysis showing that R286A has significantly lower binding than H287A (Fig. 1c) for the FKH consensus.

To identify any differences in the genomic distribution of the binding regions, CEAS was used to analyze the location of the binding peaks in the WT and DBD FOXM1 mutants (Fig. 4c). Confirming earlier results (Fig. 3d), the majority of WT FOXM1 peaks were observed to be in promoter/5′ UTR regions (63.6 % compared to genome average of 2.8 %). In both the H287A and R286A mutants, there was an increase in peaks located in the promoter/5′ UTR regions (66.8 and 81.9 %, respectively), which was more evident in the binding sites common to both mutants (86 %). These data suggest that the DBD mutant binding is preferentially retained in these regions and is also more effectively competed at other sites by endogenous WT FOXM1 protein. It is notable that the majority (80–90 %) of WT FOXM1 peaks are almost completely absent in the DBD mutant binding peaks. Figure 4d shows the ChIP-seq enrichment profiles for WT, R286A and H287A FOXM1 at three representative known FOXM1 promoter binding sites with binding peaks only being detected in the WT sample.

To determine whether any significantly enriched motifs are present in the peaks where DBD mutant FOXM1 binding is retained, MEME analysis was performed (Fig. 4e; Additional file 1: Table S4). For WT GFP-FOXM1, enriched motifs were similar to the earlier dataset (Fig. 3b) including the FKH and CCAAT box motifs. When the enriched motifs present uniquely in the WT GFP-FOXM1 were compared with those retained in the H287A and H286A mutants, it was found that the CCAAT box was common to all, while the FKH motif was only enriched in WT. This confirmed that the binding peaks retained in both the FOXM1 DBD mutants are not regions enriched in the FKH consensus. Overall, these data indicate that FOXM1 binding requires a functional DBD but the targets are in fact quite divergent, encompassing both canonical FKH and non-consensus DNA sites.

**Fig. 5:**
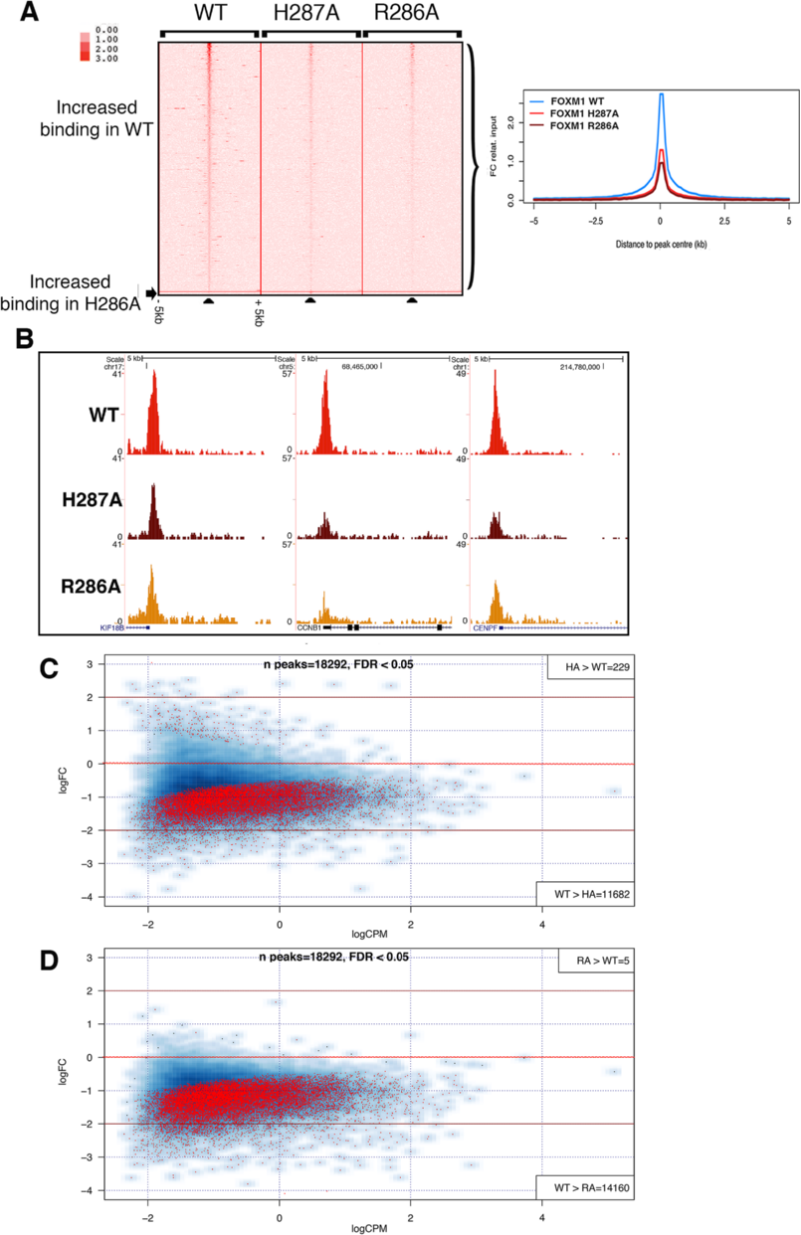
Binding of the GFP-FOXM1 DBD mutants is significantly reduced genome-wide. **a** Heat map comparing binding events in WT FOXM1 and both FOXM1 DBD mutants. The window represents ± 5 kb regions centered on WT GFP-FOXM1 binding events with a second plot (on right) showing the average signal intensity of differential bound peaks. **b** Three examples of genomic regions showing binding peaks where binding is reduced in the GFP-FOXM1 DBD mutants compared to the WT. (**c** and **d**) Differential binding analysis (DBA) was used to identify significantly (FDR <0.05) differentially bound peaks in the WT GFP-FOXM1 compared to H287A DBD mutant or the R286A DBD mutant GFP-FOXM1 cell lines. The red dots represent peaks where FOXM1 binding is significantly increased/decreased compared to the WT

It is striking that genomic sites where DBD mutant binding is still retained are predominantly those with the most significantly enriched binding in the WT GFP-FOXM1 set. For example, 77 % of the joint H287A/R286A peaks are within the top 1000 FOXM1 WT peaks (13 % of all FOXM1 WT peaks) ranked by FDR. This suggests that mutation of key DBD residues ablates binding to lower affinity sites and that only higher affinity sites are retained with the DBD mutant proteins. This phenomenon is further illustrated in the heat map in Fig. 5a, where the average signal intensity for all the binding events for each protein is shown aligned to the WT GFP-FOXM1. It can be seen that the highest intensity WT GFP-FOXM1 binding events are preserved in both DBD mutant ChIP-seq datasets, although with an overall loss in the average signal density. We also confirmed this observation by ChIP-PCR for three known promoter binding sites of FOXM1 (Additional file 1: Figure S9). Figure 5b illustrates three examples of differential binding of the DBD mutant proteins compared to the GFP-WT FOXM1 at high-affinity genomic binding sites.

To investigate whether any particular functional processes are associated with binding regions retained in the mutant DBD FOXM1 samples, the web-based tool GREAT (Genomic Regions of Enrichment Annotations) [43] was next employed. Output from GREAT identified a significant enrichment in cell cycle processes, particularly those related to M phase control (Additional file 1: Table S5), which correlates with the established functions of FOXM1 in cell cycle regulation [2]. A similar analysis on regions bound only by WT GFP-FOXM1 and not the DBD mutants showed that there was enrichment of processes associated with nucleosome organization and chromatin structure, which is consistent with the identified roles of Forkhead family members in chromatin remodeling [44]. Enrichment of processes associated with epigenetic regulation of gene expression was also revealed, which is consistent with the known link of FOXM1 to the indirect regulation of methylation through DNMT3b recruitment [39] or via HELLS expression [40]. Several other enriched processes related to translation were also identified in the GFP-FOXM1 WT dataset (Additional file 1: Table S5).

These data suggest that high affinity FOXM1 binding sites are retained in the DBD mutants and are associated with its key functions as a regulator of the cell cycle, whereas the sites where binding is lost from the DBD mutants reflect regions associated with other roles such as chromatin remodeling and epigenetic regulation. The absence of FKH motif enrichment in the DBD mutants provides strong evidence for alternative mechanisms of FOXM1 recruitment possibly mediated by indirect tethering.

To identify any statistically significant regions of differential binding between FOXM1 WT and DBD mutants, differential binding analysis (DBA, see Materials and methods) was performed. For this analysis all peaks identified in each stable cell line were first used to generate a consensus dataset containing 18,292 peaks. In the H287A mutant compared to the WT, 11,682 peaks were identified with decreased binding, and 229 peaks with increased binding (FDR <0.05) (Fig. 5c and Additional file 3). A similar analysis of the R286A mutant identified 14,160 regions with decreased binding compared to the WT and only five with increased binding (Fig. 5d and Additional file 3). No enrichment of biological processes associated with the 229 peaks with increased binding was found. These data further confirm that the majority of FOXM1 genomic binding sites are dependent on the presence of a functional DBD. The R286A mutation, which showed no discernable DNA binding in the FP assays (Fig. 1b), showed the greatest reduction in binding, (77 % of peaks significantly reduced, FDR <0.05). In keeping with the requirement of the DBD for binding, less peaks (64 %) showed decreased binding in the H287A mutant, which is consistent with the residual DNA binding activity determined by FP.

Overall, our data support the observations of Chen *et al.* [20] who also identified a set of FOXM1 binding sites lacking the FKH consensus motif, where recruitment was mediated through binding to the MuvB complex. However, our study additionally demonstrates that conserved amino acids of helix H3 in the DBD are nonetheless required for DNA binding even at non-FKH containing sites. For example, Fig. 5a illustrates that binding intensity is reduced in both DBD mutants compared to the WT even at highest intensity sites in the WT. Taken together our observations support two concurrent mechanisms for FOXM1 binding: the first being direct interaction of FOXM1 with target DNA occurring at the majority genomic binding sites, and second being recruitment or stabilization by chromatin bound proteins such as MuvB. As peaks missing in the DBD mutants include both FKH and divergent motifs, this strongly suggests that FOXM1 binding does not occur exclusively at consensus FKH sites. We further discuss these motifs further below.

### RIME proteomic analysis of WT and DBD mutant GFP-FOXM1

**Fig. 6:**
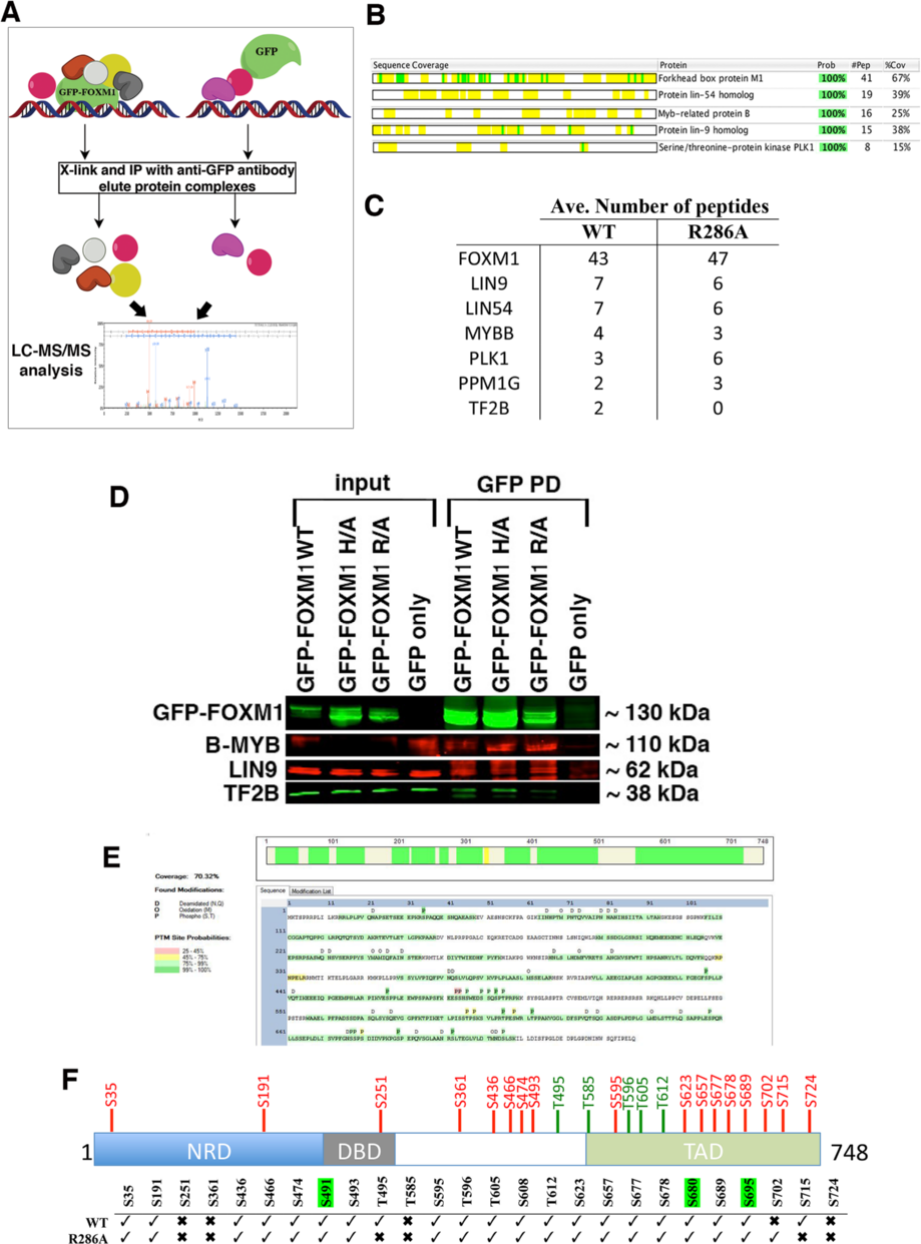
Proteomic analysis shows that the FOXM1 DBD mutants bind to same proteins as the WT. **a** Schematic diagram showing RIME analysis to identify FOXM1 co-binding proteins. **b** Coverage of GFP-FOXM1 and associated high-confidence interacting proteins. Yellow shading indicates regions of peptide coverage and the green shading shows post-translational modifications identified. (Prob = probability, # pep = number of peptides, %Cov = % protein coverage). **c** Table showing the average number of peptides identified for the six top proteins present in the WT GFP-FOXM1 pull-downs. **d** Co-immunoprecipitation showing pull-down of B-MYB, LIN9, and TF2B with a GFP antibody in extracts from HEK293 cells expressing WT, H287A, and R286A GFP-FOXM1 DBD mutants and GFP only. **e** Schematic diagram showing phosphorylation sites identified by Proteome viewer in a WT GFP-FOXM1 RIME sample. The color indicates the identification probability. **f** Diagram showing the position of previously identified phosphorylation sites in FOXM1b (red indicates serine and green threonine residues). The table indicates sites identified from the RIME analysis in WT and R286A DBD mutant GFP-FOXM1, with novel sites highlighted in green

To investigate whether the reduced DNA binding of the DBD mutant proteins is due to perturbation of protein-protein interactions involved in the recruitment, proteomic analysis of the FOXM1 interacting proteins was performed by RIME (Rapid immunoprecipitation mass spectrometry of endogenous proteins) [45] for both the WT GFP-FOXM1 and the R286A DBD mutant. Formaldehyde-fixed chromatin was isolated from HEK293 cells expressing either GFP only, GFP-FOXM1 WT, or GFP-FOXM1 R286A and immunoprecipitation was performed with an anti-GFP antibody followed by LC/MS-MS (Fig. 6), to identify associated proteins. To eliminate any non-specific interactions with GFP, which can occur through cross-linking of the GFP to highly abundant proteins such as keratins and ribosomal sub-units, proteins identified in the GFP only expressing cell line were not considered in the WT or R286A samples. High confidence interacting proteins were then identified using the Scaffold proteomic analysis tool [46], details of the proteins identified are shown in Additional file 4. In the WT, several proteins previously shown to interact with FOXM1 were identified, including components of the MuvB complex [19] (LIN9, LIN54), B-MYB [47] and PLK1 [30]. Figure 6b shows the sequence coverage from Scaffold for the top five high confidence proteins. These results show that the presence of the GFP epitope tag does not significantly affect the interaction of the GFP-FOXM1 fusion protein with known partners of endogenous FOXM1. Overlap of proteins identified in the WT and R286A replicates (identified by unique peptides present in at least three out of the four samples) revealed 21 common to both, while eight were present in the WT only and 15 in the R286A mutant (Additional file 1: Table S6 and Figure S10).

When the highest confidence interacting proteins identified in the WT were compared with those in the R286A DBD mutant (Fig. 6c), it was of note that the FOXM1 DBD mutant was still able to interact with MuvB components (LIN9, LIN54), PLK1 and B-MYB. This interaction was further confirmed by co-IP for GFP-FOXM1 with B-MYB and LIN9 (Fig. 6d). We observed that the general transcription factor TF2B, which is known to directly interact with FOXM1 [48], was identified in all the WT FOXM1 RIME samples, however for the DBD mutants T2FB was seen in only one of four replicates of the R286A mutant. It is possible that the inability of the DBD mutant to bind DNA impairs the assembly of active transcriptional complexes despite preserving the direct binding of core FOXM1 interacting proteins.

In both the WT and DBD mutant RIME samples, other components of the MuvB complex [19], LIN37 and LIN54 were also identified, albeit at lower confidence (Additional file 1: Table S6). The identification of these known FOXM1 binding proteins in the R286A mutant, which show reduced chromatin recruitment, could be due to interaction with nuclear non-chromatin bound FOXM1. Other proteins identified include the serine/threonine phosphatases PPM1G and PP1G; and it is of note that a subunit of the related phosphatase PP2A is known to interact with FOXM1 [49], while PPM1G is thought to have a key role in cell cycle regulation and in the DNA damage response [50]. Analysis of the biological pathways associated with the WT GFP-FOXM1 interacting proteins using the GeneGo Metacore tool (Additional file 1: Figure S11), showed a range of processes that correlate with known FOXM1 roles including cell cycle, DNA damage and nucleosome assembly.

FOXM1 activity is regulated by a series of CDK-cyclin mediated phosphorylation steps [17, 51], which are essential for the formation and nuclear localization of the transcriptionally active protein at the correct stage of the cell cycle [17, 52]. We therefore compared serine and threonine phosphorylation modifications present in both the WT and R286A DBD mutant FOXM1 proteins isolated by GFP pull down and subjected to RIME. Analysis using the Proteome Discoverer with the PhosphoRS algorithm [53] revealed the presence of many previously described phosphorylation sites that are required for FOXM1 transactivation (Fig. 6e and f; Additional file 1: Figure S12 and Table S7; Additional file 5). These data also show that the R286A mutant protein undergoes a similar pattern of phosphorylation as the WT protein. Our analysis identified one of two known PLK1-mediated serine phosphorylation sites (S715 but not S724) in the transactivation domain (TAD) of FOXM1 [30]. It is unclear why this phosphorylation event was only seen in the WT and not the R286A mutant and this might suggest that phosphorylation happens after FOXM1 has bound chromatin. In addition, this analysis identified several novel serine phosphorylation sites present in both WT and mutant FOXM1, including S608 and S680 (Additional file 1: Table S7), however their biological relevance is unclear at present.

Overall, RIME has confirmed previously known protein interactions and identified novel FOXM1 protein interactions and post-translational modifications. The majority of known and novel phosphorylation marks were found in both mutant and WT protein, with the notable exception of PLK1 target S715. Furthermore, the significant reduction in DNA binding for the R286A mutant protein shown in the ChIP-seq analysis is not due to this mutation affecting protein-protein interactions or phosphorylation, as overall similar profiles to the WT were observed.

### FOXM1 DBD mutations inhibit expression of FOXM1-regulated genes

**Fig. 7:**
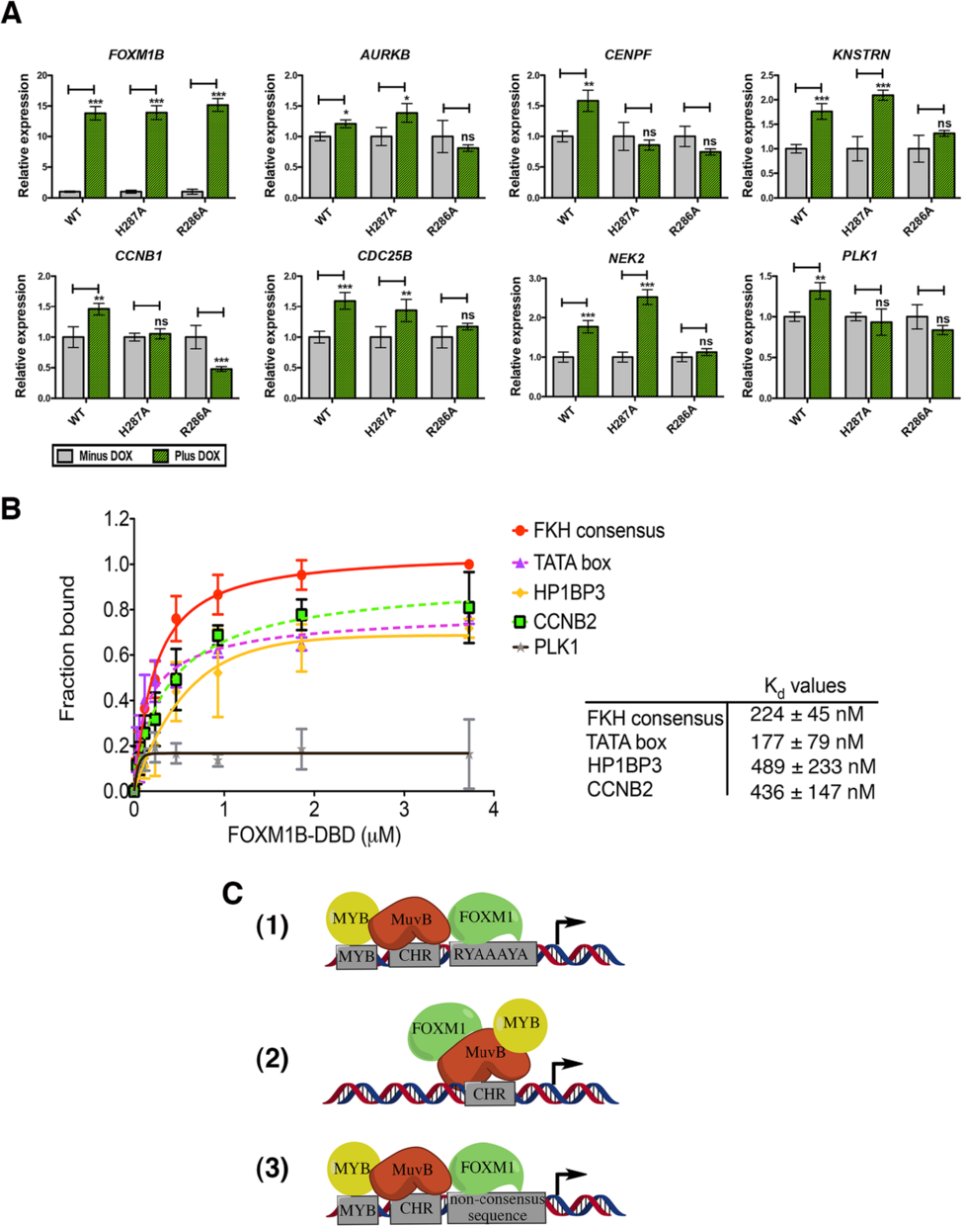
FOXM1 transcriptional activity requires direct chromatin interaction involving recruitment to non-consensus sequences. **a** qPCR analysis of the mRNA transcript levels in GFP-FOXM1 WT or mutant (H287A or R286A) cell lines treated ± doxycycline (1 μg/mL) for 24 h showing the relative change in the levels of *FOXM1B*, *AURKB*, *CCNB1*, *CDC25B*, *CENPF*, and *PLK1*. In each case the data are normalized to the minus doxycycline control. **b** Binding curves measured by fluorescence polarization analysis (assay details in the Materials and methods section), showing binding affinity of GST-FOXM1B DBD for 16-mer [FAM]dsDNA sequences present in FOXM1 binding peaks from the ChIP-seq dataset compared to the FKH consensus. The plot shows the fraction bound with increasing protein concentration. The table shows the *K*_d_ values ± SD determined for each sequence. **c** Illustration of alternative models proposed the recruitment of FOXM1 to chromatin. (1) Direct DNA binding of FOXM1 at promoter sites containing a FKH consensus motif and interaction with MuvB and B-Myb. (2) FOXM1 is recruited by MuvB complex and does not directly bind to the DNA. (3) FOXM1 binds directly at non-consensus sequences facilitated by interaction with MuvB and B-Myb. Arrow indicates transcription start site of target gene. Data representative of triplicate experiments ± SD. (*) *P* <0.05, (**) *P* <0.01, (***) *P* <0.001

To investigate the effect of the DBD mutations on FOXM1-regulated gene expression, qPCR was used to measure the expression levels of several known target genes (Fig. 7a). Transcript levels were compared in the WT and DBD mutant cells (H287A and R286A) between ± doxycycline. The level of GFP-FOXM1B induction was first measured to ensure comparable expression of the WT and DBD mutants. Indeed by qPCR, the addition of doxycycline addition led to an approximately 13-fold increase in GFP-FOXM1 expression in both the WT and DBD mutant cell lines compared to the non-induced WT GFP-FOXM1 cells (Fig. 7a). Next, the expression of seven known FOXM1 target genes was measured; *AURKB*, *CENPF*, *KNSTRN*, *CCNB1*, *CDC25B*, *NEK2*, and *PLK1.* In each case, a significant increase in expression was observed in the GFP WT-FOXM1 cell line following induction by doxycycline (*P* <0.05). Although the relative increase in expression was small (in the range of 1.3-fold for *AURKB* to 1.8-fold for *NEK2*), this is in the context of endogenous FOXM1 and comparable to previous studies [3, 31]. In contrast, for the R286A DBD mutant there was no significant difference in transcript levels following induction, whereas for the H287A DBD mutant, three transcripts (*CENPF*, *CCNB1*, and *PLK1*) showed no significant change on induction This latter result may reflect a higher residual level of DNA binding activity that is observed in the FP assay and by ChIP-seq when compared to R286A. This is supported by inspection of the ChIP-seq data for three transcripts (*KNSTRN*, *CDC25B*, and *NEK2*) that show different responses in the H287A compared to the R286A mutant (Additional file 1: Figure S13). In each case the peak in the promoter region of the gene is significantly smaller in the R286A sample.

These data show that mutations in the DBD, which significantly impair the DNA binding interaction of FOXM1, reduce the induction of downstream target genes. The reduction in transcription seen in the R286A DBD mutant correlates with RIME analysis (Fig. 6c and d) showing a decreased association with the general transcription factor TF2B, which forms part of the RNA pol II pre-initiation complex [54]. The R286A DBD mutant reduces expression to levels seen in the uninduced cells, which suggests that it is unable to block the transcription mediated by endogenous FOXM1 protein binding.

### FOXM1 binds to lower affinity consensus sequences

Our ChIP-seq results show that overall FOXM1 binding is significantly depleted when the DNA binding affinity is reduced, demonstrating the requirement of a functional DBD. However, only approximately 14 % of the total binding peaks in the WT FOXM1 contain a canonical FKH consensus sequence suggesting that FOXM1 DBD must also be required for recruitment to non-consensus motifs. FOXM1 binding to alternative motifs has also been supported by high-throughput studies which show that Forkhead factors (including mouse Foxm1) bound two distinctly different DNA sequence motifs [55]. Furthermore, when the Systematic Evolution of Ligands by EXponential Enrichment (SELEX) method was combined with ChIP-seq, a greater diversity of FKH binding sites was also identified [56]. To confirm whether FOXM1 binds directly to non-consensus sequences identified by ChIP-seq, FP assays were used to determine the *in vitro* binding affinity of GST-FOXM1 for non-canonical DNA sequences (Fig. 7b). Regions highly enriched in FOXM1 binding at gene promoters were selected from the ChIP-seq dataset, using sequences located at the peak center with partial similarity to the FKH motif (enriched in A/T bases). Additionally, a previously reported non-consensus FOXM1 binding site in TATA box of *MYC* [57] was included. As expected the FOXM1B DBD associates with a positive control FKH consensus sequence in a dose dependent manner (*K*_d_ of 224 ± 45 nM). The FOXM1B DBD also associates with several non-consensus sequences tested including CCNB2 (*K*_d_ = 436 ± 147 nM), HP1BP3 (*K*_d_ = 489 ± 233 nM), and the MYC TATA box (*K*_d_ = 177 ± 79 nM), but only weakly with the others such as PLK1 where a *K*_d_ value could not be determined. Similar results were observed by Electrophoretic Mobility Shift Assay (EMSA) (Additional file 1: Figure S14) although the apparent binding affinities measured were lower (*K*_d_ of 346 ± 24 nM for FKH consensus).

Collectively, these results together with previous *in vitro* binding studies [55, 58] demonstrate that FOXM1 can bind to DNA sequences with significant sequence divergence from the canonical FKH motif. Furthermore, our results using ChIP-seq and expression analysis with FOXM1 DBD mutants show that recruitment to non-consensus sites is also critical for transcriptional activity. Overall, our data support a mixed model of direct FOXM1 recruitment to chromatin at lower affinity non-consensus sequences mediated by protein-protein interactions.

## Discussion

The aim of this study was to investigate the mechanisms regulating FOXM1 DNA binding in a cellular context. FOXM1 has previously been demonstrated to bind to the FKH consensus motif *in vitro.* In common with other Forkhead factors, this interaction is mediated by the DNA binding domain [57], although with lower affinity compared to other FKH factors [59]. Since FOXM1 has also been suggested to bind at non-FKH consensus sequences mediated by indirect protein-protein interactions, we examined the role of direct versus indirect DNA binding in FOXM1 recruitment using GFP-tagged FOXM1 expressed in HEK293. Exogenous expression of a GFP epitope-tagged protein enabled the generation of cell lines with specific point mutations in the DBD of FOXM1 for use in ChIP-seq analysis while avoiding the known phenotypic responses caused by reduced FOXM1 transactivation [60], such as inhibition of the cell cycle and mitotic catastrophe.

Results for GFP-FOXM1 WT showed that <14 % peaks contained a consensus FKH motif, which is consistent with previous results investigating endogenous FOXM1 binding in U2OS cells [20]. However, in contrast to the prior study, our results provide strong evidence that DNA recognition by the FOXM1 DBD remains critical for recruitment to non FKH-consensus genomic binding sites as opposed to an indirect mechanism mediated solely by protein-protein interactions. Our results suggest that FOXM1 binding occurs by a process of assisted recruitment, as proposed by Rabinovich *et al.* [61] for the E2F transcription factor, in which most binding sites lack a consensus motif with binding mediated by additional transcription factors at lower affinity DNA target sites. Indeed, genome-wide studies have highlighted several transcription factors that show low enrichment of the consensus sequence in their binding sites [61], suggesting that this model of assisted recruitment is a more general mechanism of transcription factor binding.

Two models previously proposed for the assembly of FOXM1 with the MuvB complex and B-Myb protein on cell cycle-regulating promoters are shown in Fig. 7c. The first (1) shows FOXM1 binding to FKH consensus sequences in concert with recruitment of the MuvB complex present at cell cycle homology (CHR) sequences and B-Myb bound at a MYB binding site in close proximity [19]. In the second (2), FOXM1 is indirectly recruited by the MuvB complex at CHR sites together with B-Myb, without contribution of DBD-DNA interactions [20]. A third model (3) that is inferred by our results shows an alternative, mixed mode for FOXM1 recruitment. This mechanism involves a degree of DNA binding at lower affinity, non-canonical sites that is also facilitated by protein-protein interactions. This latter model would account for the lack of FKH motifs in the peaks enriched in FOXM1/FOXM1-GFP pull-down, and is supported by our data showing that mutations of the DBD in FOXM1 significantly reduce genome-wide binding at both consensus and non-consensus sites without affecting either the protein-protein interactions or the phosphorylation status of FOXM1.

Considering ours and previous biophysical binding data, it is probable that FOXM1 exhibits some degree of DNA binding to the consensus FKH motif. Indeed, we observe that while FOXM1/FOXM1-GFP ChIP-seq reveals a majority of peaks at non-FKH motifs, the FKH motif is still apparent present in a small but significantly enriched set (14 %, *P* = 10^−144^). Future experiments such as genetic deletion of discrete motifs by CRISPR would be needed to unambiguously establish which FKH or other motifs FOXM1 directly associates. Nonetheless, our data support the previously proposed model 1 as well as that suggested by model 3, namely that FOXM1 binding in chromatin operates through a mechanism dependent on a functional DBD assisted by local protein-protein recruitment regardless of sequence content. In support of this, mutant FOXM1 is unable to induce transactivation of known FOXM1 target genes, all of which lack a canonical FKH consensus within the FOXM1 binding site. It is also notable that binding studies with WT FOXM1 confirmed that the protein additionally binds non-consensus sequences, further supporting an assisted model of FOXM1 binding whereby protein recruitment stabilizes the association.

Besides an additional model of FOXM1 chromatin recruitment, our work has also revealed novel protein-protein interactions of FOXM1 by use of the RIME methodology [45]. Newly identified FOXM1-interacting proteins include two phosphatases, PPM1G and PP1G, which may act in a similar manner to the PP2A phosphatase that regulates FOXM1 activity during the cell cycle by controlling dephosphorylation to prevent premature transcriptional activity [49]. The E3 Ubiquitin-Protein Ligase, UHRF1 was identified in all the WT GFP-FOXM1 samples. UHRF1 is known to regulate gene expression and the cell cycle and is overexpressed in many cancers [62], and it is of note that UHFR1 binds inverted CCAAT motifs [63] perhaps enabling requirement of FOXM1 at these specific genomic binding sites.

The proposed model of assisted recruitment for FOXM1 (Fig. 7c, 3) could be applied more widely to include binding sites other than those at cell-cycle related promoters, particularly at known sites of MuvB complex and B-Myb binding [19, 20, 47]. This is exemplified by the binding of FOXM1 in the COX2 promoter, which is mediated by interaction with the SP1 transcription factor at the SP1 binding site in the absence of a canonical FKH motif [64]. This further suggests that binding to non-consensus sequences at intronic and intergenic sites could be facilitated by other transcription factors.

The importance of the Forkhead DBD for direct interaction with target DNA sites has been shown for a number of other Forkhead factors, with disease-causing mutations having been identified in the FKH DBD domain [65, 66]. Both DBD mutations examined in this study are known naturally occurring missense mutations associated with the loss-of-function of the Forkhead factor. For example, the R553H mutation in FOXP2 (equivalent to FOXM1 R286) is associated with development of a severe speech language disorder [67], while a mutation of the same arginine residue in FOXC2 (R121C) is associated with a developmental disorder affecting the lymphatic vasculature system. In the case of FOXM1, the fact that no similar disease-causing mutations have been associated with the DBD supports the critical importance of direct DNA binding for FOXM1 function as are likely to compromise cell viability. Indeed, Korver *et al.* [41], suggests that any loss-of-function mutations in the FOXM1 DBD would be embryonic lethal and therefore are not represented within the general population.

In fact, it is overexpression of WT FOXM1 that is associated with disease. In many human cancers FOXM1 overexpression promotes aberrant activation of FOXM1 target genes, contributing to oncogenesis and facilitating invasion, metastasis, and therapeutic resistance [6]. Evidence from this study along with results from our previous work [25, 26], showing inhibition of FOXM1 DNA binding by direct interaction with both a small molecule, FDI6 and with the natural product thiostrepton, suggests that targeting the DNA binding domain of FOXM1 might provide a particularly effective means to ameliorate such a disease phenotype. We are currently developing chemical tools to test this hypothesis in future experiments.

## Conclusions

Overall, we have demonstrated that the DNA binding domain of FOXM1, in common with other Forkhead factors is necessary for recruitment to DNA at consensus and non-consensus FKH genomic binding sites and activation of down-stream transcriptional activation. Furthermore, we found that FOXM1 DBD mutants are unable to bind DNA yet maintain similar protein-protein interactions to the WT protein. Finally, we identified novel FOXM1 phosphorylation sites and found that the majority of all phosphorylation events are unaffected by DBD mutation. The mechanism of interaction of FOXM1 with DNA is of critical importance to support any development of novel therapeutics designed to specifically target the DBD of FOXM1, and thereby reducing transactivation of FOXM1-regulated genes caused by overexpression in cancer.

## Materials and methods

### Cell culture

Human HeLa TRex and HEK293 Flp-In cells were obtained from Invitrogen and grown in EMEM supplemented with 10 % tetracycline free FBS and 5 μg/mL blasticidin (Sigma) or DMEM supplemented with 10 % tetracycline free FBS and 100 μg/mL zeocin (Invitrogen), respectively.

### Generation of FOXM1 expressing cell line

The EGFP-FOXM1B fusion plasmid was a gift from Dr M. Teh (Queen Mary University of London) and was cloned into the pcDNA4/TO plasmid (Invitrogen) for transient expression in HeLa TRex cells or into the pcDNA5FRT (Addgene) to generate stable cell lines. HEK293 Flp-In cells (Invitrogen) were first transfected with a pcDNA6/TR plasmid (Invitrogen) and selected with 5 μg/mL blasticidin to generate a stable HEK293tetR Flp-In cell line. This cell line was co-transfected with the GFP-FOXM1 and pOG44 (Flp recombinase vector) and selected with 100 μg/mL hygromycin (Invitrogen).

### Fluorescence polarization assays

Fluorescence polarization (FP) assays were performed to assess the binding of the FOXM1 mutant DBD compared to the WT, using a 16mer dsDNA forkhead consensus sequence (AAACAAACAAACAATC) labeled with carboxyfluorescein at the 5′ position on one strand. Assays were performed with serial dilutions of DBD proteins from 5 μM to 80 nM in FP-binding buffer (50 mM Tris–HCl pH 7.5, 5 mM MgCl_2_, 1 mM DTT, and 2 % glycerol). Fluorescence was measured using 485 nm excitation and 520 nm emission filters. Binding plots were generating after expressing the data as the fraction bound over protein concentration, with fraction bound defined as:

$$ \%\kern0.24em \mathrm{bound}=\frac{P-{P}_0}{P_{100}-{P}_0} $$

(Where *P*_*0*_ is the polarization value at 0 % saturation, *P*_*100*_ is the polarization value at 100 % saturation, and *P* is the observed fluorescence polarization (FP) at each concentration point.)

### Luciferase reporter assays

The pGL4.26 plasmid (Promega) containing a minimum promoter upstream of the firefly luciferase gene was used to generate a reporter containing six copies of the FKH binding consensus as described by Major *et al.* [17]. For the 6X FKH consensus, a dsDNA sequence containing 72 bp with 5′ phosphate groups was ordered from IDT (Integrated DNA technologies). For the CCNB1 and PLK1 reporters, 200 bp sequences from the CCNB1 and PLK1 promoters were synthesized and inserted into the promoter-less pGL3 luciferase vector (Promega). Sanger sequencing was performed commercially to confirm the constructs (ATGC Inc.). The renilla control plasmid, pRL-TKL, (Promega) was used to normalize for transfection efficiency. Luciferase assays were performed using the Dual Luciferase Kit (Promega) as described by the manufacturer. Briefly, HeLa TRex cells were plated at 2 × 10^4^ cells/well in 96 well plates in antibiotic free media containing 10 % tetracycline free FCS and cultured overnight. Transfections were performed using lipofectamine 2000 following the manufacturer’s protocol. For each pcDNA4/TO pEGFP FOXM1 construct replicates of 10 were prepared; each transfection contained: 50 ng/well pEGFP-FOXM1, 50 ng/well pGL4.26 (6XBD), or pGL3 CCNB1/PLK1 and 10 ng/well pRL-TK. Plates were cultured overnight, then for each condition, five wells had 100 μL culture media added and five had media plus doxycycline (2 μg/mL). These were incubated for an additional 24 h period. Luciferase activity was measured using the Dual Luciferase Kit (Promega). Luminescence readings were taken after the addition of each reagent and relative activity calculated by obtaining the ratio of firefly luciferase to renilla to account for transfection efficiency, then the ratio of plus doxycycline to minus to give the relative induction. Four independent experiments were run with five replicates for each condition. The control non-FOXM1 responsive promoter for SV40 in pGL4.10 was obtained from Promega and the human CYP1B1 promoter was cloned from human gDNA using primers containing *Acc65I/EcoRV* restriction sites generating a 600 bp product:

- Fw: GACTGGTACCGGATTCCTGATCTCGCCGCAAGAACTGG
- Rv: GACTGATATCCGTTGAGATTGAGACTGGGGGTCGG

The PCR product was digested and ligated into the pGL4.10 vector (Promega).

### Western blots

Western blots were performed using antibodies for anti-FOXM1 (sc-502) and anti-HA (sc-543) and anti-β-actin (ab6276) purchased from Abcam. Fluorescent imaging of GFP-FOXM1 was performed using an anti-GFP antibody (ab290) purchased from Abcam. Cell lysates were prepared using RIPA buffer (20 mM Tris–HCl, pH 7.5, 150 mM NaCl, 1 mM Na_2_EDTA, 1 mM EGTA, 1 % NP-40, 1 % sodium deoxycholate, 2.5 mM sodium pyrophosphate, 1 mM ß-glycerophosphate, 1 mM Na_3_VO_4_) with proteasome inhibitors (Roche). Lysate was agitated for 30 min at 4 °C by end-to-end rotation, supernatant collected following centrifugation (13,000 rpm, 4 °C, 10 min) and protein concentration measured by BCA (Pierce) assay. Samples loaded onto 4–12 % Tris-Glycine mini gels (Invitrogen), purified by SDS-PAGE and transferred to nitrocellulose membrane (Invitrogen). Membranes were incubated in Odyssey blocking buffer (LiCor) for 1 h at room temperature and probed with FOXM1, HA, or GFP antibody 1:1,000 and β-actin 1:5,000 overnight at 4 °C. For detection, the blot was incubated with LiCor IRDye secondary antibodies; 680LT goat anti-rabbit IgG and 800LT goat anti-mouse IgG both at 1:10,000 and visualized using an Odyssey scanner.

### Immunofluorescence

Cells were plated 4 × 10^4^/well in Ibidi treated 8-well culture slides (Ibidi) and left to adhere overnight. Following doxycycline treatment for 24 h the cells were washed 1X with PBS and fixed with 4 % methanol-free formaldehyde at room temperature for 15 min. After washing 3X with PBS, cells were permeabilized and blocked using blocking buffer (PBS/5 % normal goat serum/0.3 % Triton-X 100) for 1 h at room temperature. GFP antibody was diluted in antibody blocking buffer (PBS/1 % BSA/0.3 % Triton-X 100) FOXM1 (1:500) and added to appropriate wells leaving control wells with buffer only. Culture wells were incubated at 4 °C overnight followed by washing 3X with PBS. Secondary antibodies were diluted 1:2,000 in antibody dilution buffer, using either goat anti-Rabbit Alexa Fluor 488 (Invitrogen) and added for 1 h in the dark at room temperature. Culture wells were washed 3X with PBS, with one wash containing DAPI (1 μg/mL) for nuclear staining. Liquid was removed from wells and anti-fade mounting media (Ibidi) added to each well. Plates were stored in the dark at 4 °C and visualized using an inverted Leica DMI6000B microscope.

### Quantitative real-time PCR analysis

RNA was collected after the indicated time-points and qPCR was performed using Power Sybr mix (ABI) on a CFX96 Real-time thermal cycler (Bio-Rad). Total RNA was extracted using the RNeasy mini kit (Qiagen) following the manufacturer’s protocol. cDNA was prepared from 1 μg RNA using Maxima reverse transcriptase (Fermentas) following the manufacturer’s protocol. qPCR was performed in triplicate in 10 μL reactions with Power sybr mix (ABI) using Qiagen quantitect primers for *B2M*, *ACTB*, *CCNB1*, *CDC25B*, and *FOXM1* and additional primers shown below. *ACTB* and *B2M* were used as housekeeping genes for normalization of the data. PCR conditions were: 95 °C 10 min, 40 cycles of 95 °C for 15 s, and 60 °C for 30 s followed by a dissociation curve (60–95 °C). Relative expression levels were calculated using the delta delta C_T_ method [68].

### Chromatin immunoprecipitation

ChIP experiments were performed as previously described [69] using the following antibodies: anti-FOXM1:Genetex (GTX1000276), Genetex (GTX102170), and anti-GFP (Abcam ab-290). Experimental details and primer sequences are listed below.

**Primer sequences:**

**qPCR primers**

**Table:** 

**Primer name**	**Forward**	**Reverse**
AURKB	TACGGCCGACAGACGGCTCCA	AGCGGCTCATGAGGACAAGTGC
CENPF	CGGCTGCGGGCAGTTTGAAT	AAATAAACTTGCTCTCGGGGACG
FOXM1A	TGGGGAACAGGTGGTGTTTGG	GCTAGCAGCACTGATAAACAAAG
FOXM1B	CCAGGTGTTTAAGCAGCAGA	TCCTCAGCTAGCAGCACCTTG
FOXM1C	CAATTGCCCGAGCACTTGGAATCA	TCCTCAGCTAGCAGCACCTTG
FOXM1 UTR	TCCCTGCTGCCTGATTATGC	TCACCATTGCCTTTGTTGTTC
KNSTRN	CCCTGGCATCACGACAAGAA	TCCAAGCAATCTGTAACTCCTCC
NEK2	GTCTCCTGAACAAATGAATCGC	CTCATACAGCAAGCAGCCCA
PLK1	TTCCCAAGCACATCAACCCCGT	AATGGTTGGGCGGGCAGTGG

**ChIP-qPCR**

**Table:** 

**Primer name**	**Forward**	**Reverse**
AURKB promoter	GGGGTCCAAGGCACTGCTAC	GGGGCGGGAGATTTGAAAAG
CCNB1 promoter	CGCGATCGCCCTGGAAACGCA	CCCAGCAGAAACCAACAGCCGT
CDC20 promoter	TCTCGTGATAGCTGAGACTTTCC	CTATTGGCTCCTTCAAAATCCA
CDC25B promoter	AAGAGCCCATCAGTTCCGCTTG	CCCATTTTACAGACCTGGACGC
CDK1 promoter	TAGCCGCCCTTTCCTCTTTC	CAAAGCAGCCAATCAGCGA
CDKN3 promoter	AGCCAATCAACGTCAACACAG	GACTCGGCCTCTAATCGCTG
CENPF promoter	CACCTCCAGTAGAGGGGCTTG	TACCTCCACGCCTATTGGTC
KIF20A promoter	TCTGATTGGCCGAACGAACG	TACTCACACCTAGTCGGCGA
NEK1 promoter	GTTTGGAAGGGCAAAGGAAT	GTCACAGAGAGGTTTGGGAGTAA
PLK1 promoter	CCAGAGGGAGAAGATGTCCA	GTCGTTGTCCTCGAAAAAGC
TOP2A promoter	CGGAAAGCTTGGAAGAGATG	AGATTGGCAGTTCCTGGAGA
Actin control	AGCGCGGCTACAGCTTCA	CGTAGCACAGCTTCTCCTTAATGT
Cyclin D1 Control	TGCCACACACCAGTGACTTT	ACAGCCAGAAGCTCCAAAAA

**ds[6FAM] sequences used for FP and EMSA analysis**

**Table:** 

**Primer name**	**Top strand**	**Bottom strand**
Consensus	[6FAM]AAACAAACAAACAATC	GATTGTTTGTTTGTTT
HP1BP3	[6FAM]CCTCAGCCAATCGGGG	CCCCGATTGGCTGAGG
PLK1	[6FAM]TCGGGAGCATGAGTGC	GCACTCATGCTCCCGA
CCNB2	[6FAM]ACGCGGTATTTGAATC	GATTCAAATACCGCGT
CCNB1	[6FAM]GAACCTTTTGAAAAAG	CTTTTTGAAAAGGTTC
MYC P2 TATA box	[6FAM]TGAGTATAAAAGCCGG	CCGGCTTTTATACTCA
CDK1	[6FAM]GCTGCTTTGAAAGTCT	AGACTTTCAAAGCAGC

### ChIP-sequencing experiments

Initial ChIP-Seq experiments were performed using two biological replicates for both endogenous and GFP-tagged FOXM1 in HEK293 cells. To analyze the WT GFP-FOXM1 versus DBD mutants, three replicates were performed for the WT and H287A and two for the R286A HEK293 stable cell lines (Additional file 1: Table S1). ChIP DNA was processed for Illumina sequencing as previously described [69]. Further details are given in Additional file 3. Data are available through the NCBI’s Gene Expression Omnibus [70] using GEO Series accession number GSE60032. Single end 36-bp ChIP-seq data were generated by the Illumina analysis pipeline CASAVA 1.7 and OLB 1.9.4. Reads were aligned to the Human Reference Genome (assembly hg19, NCBI Build 37, February 2009) using bwa 0.6.1 [71] with default settings and reads that could not be confidently assigned to a unique genome position (that is, with mapping quality mapq <15) were removed (Additional file 1: Table S1). In addition, reads overlapping regions known to accumulate unusually large number of reads in a non-specific manner were excluded (excluded regions obtained from [72]). Read-enriched regions (that is, binding sites) were identified with MACS 1.4.1 [34] using as control file a genomic input prepared from the same cell lines as the ChIP libraries.

### RIME analysis

RIME analysis was performed as previously described [45] using the anti-GFP antibody (ab290, Abcam). Samples were processed for LC/MS-MS and analyzed by the Proteomics Core facility at the CRUK Cambridge Research Institute. The RIME protocol (Rapid Immunoprecipitation Mass Spectrometry of Endogenous proteins) developed by Mohammed and Carroll [73] was used to identify FOXM1 interacting proteins. Preparation of nuclear fraction is similar to that described for ChIP samples in with minor modification: cells from 4 × 15 cm^2^ dishes were cross-linked using 1 % methanol-free formaldehyde (Pierce) for 7 min and quenched with 2.5 M glycine (final concentration 0.2 M). After scraping the plates the cells were combined a 15 mL tube and lysed with 10 mL LB1, 10 mL LB2, then resuspended in 1,200 μL LB3 and split into 4 × 1.5 mL tubes for sonication. Protein G Dynal beads (Invitrogen) were used for the IP and after overnight incubation with the cleared lysate the beads were washed 10X with RIPA buffer followed by 2X with 100 mM ammonium hydrogen carbonate solution. Following the first wash, the beads were transferred to new tubes. Antibody used for pull-down was GFP (Abcam; ab290). Experiments were performed in triplicate.

Samples were processed and analyzed by LC-MS/MS by the proteomics core at the CRUK Cambridge Institute. In brief, all samples were analyzed using MASCOT (Matrix Science, London, UK; version 1.3.0.339) and X! Tandem (The GPM, thegpm.org; version CYCLONE (2010.12.01.1)) protein identification software. Mascot was set up to search Mascot5_SwissProt_Homo sapiens (human) (unknown version, 20,284 entries) assuming the digestion enzyme trypsin. X! Tandem was set up to search a subset of the SwissProt_2013_05 database also assuming trypsin digestion. Mascot and X! Tandem were searched with a fragment ion mass tolerance of 0.80 Da and a parent ion tolerance of 10.0 PPM. Deamidation of asparagine and glutamine and oxidation of methionine were specified in Mascot as variable modifications. Glu- > pyro-Glu of the n-terminus, ammonia-loss of the n-terminus, gln- > pyro-Glu of the n-terminus, deamidation of asparagine and glutamine and oxidation of methionine were specified in X! Tandem as variable modifications.

Protein identifications were accepted if they could be established at greater than 99.0 % probability based on the peptide coverage and contained at least one unique peptide. Protein probabilities were assigned by the Protein Prophet algorithm [74]. Phosphorylation sites were identified with Proteome discoverer (Thermo Fisher Scientific).

### Analysis of differential binding

To identify regions of differential FOXM1 binding between the WT and DBD mutant samples, a general linear model was fitted to each putative binding site to test for the difference in read count between treatments. Model fitting and testing was performed using the Bioconductor library edgeR [75] using the function estimateGLMTagwiseDisp for estimating the dispersion parameter of the negative binomial distribution and glmFit and glmLRT for fitting and testing the difference of treatment of each binding site [76]. The heatmaps were prepared with R. Hierarchical clustering was performed using the hclust package in R.

### Motif analysis and genomic distribution of binding events

The *cis*-regulatory element annotation system (CEAS) [37] function in cistrome (http://cistrome.org/ap/) was used to functionally annotate binding sites. Known transcription factor motifs significantly enriched in the binding sites were identified with MEME suite [77]. Transcription factor motifs enriched in the sequences spanned by the ChIP-Seq peaks have been discovered with ame program [78]. Transcription factor motifs were obtained from the JASPAR database included in the meme suite [79]:

Where $infasta is the FASTA file of sequences spanning each ChIP-Seq peak. The significance threshold for motif detection was set to the default value of 0.05.

Percentages for the enriched motifs were calculated by matching peak sequences to the following regular expressions, where letters are according to the IUPAC nucleotide code: TTTRAAW (CHR); GGGMGGGR (SP2); CCAATSR (NFYB); RTAAAYA (FOXP2); AGRDGGCG (CTCF); YTTCCGG (ELK4).

### GO pathway analysis

GO pathway enrichment was performed using GeneGo metacore (MetaCore from Thomson Reuters) and visualized with REViGO (Reduce and Visualize Gene Ontology) [38].

### Co-immunoprecipitation

Experiments were performed using the nuclear co-IP kit from Active motif with pull-down using a FOXM1 antibody (Santa Cruz sc-502) following the manufacturer’s protocol with immunoprecipitation carried out using the low buffer provided supplemented with 1X protease inhibitor cocktail). Detection was performed by western blotting using B-Myb (Santa Cruz sc-724), Lin9 (Abcam ab-71887), and TFIIB (Santa Cruz sc-225) with LiCor IRDye secondary antibodies (800LT goat anti-mouse, 680LT goat anti-rabbit and 680LT donkey anti-goat). Cells were harvested at 70 % confluence in 15 cm^2^ dishes using PBS with phosphatase inhibitors at 4 °C and spun at 1,500 rpm for 5 min at 4 °C. Nuclear lysates were prepared by resuspending the cell pellets in 1X hypotonic buffer and incubating for 15 min on ice, following detergent addition the supernatant was centrifuged at 14,000 ×g for 30 s to pellet the nuclear fraction. The nuclear fraction was digested using complete digestion buffer with addition of the enzymatic shearing cocktail at 4 °C for 2 h with end-to-end rotation. EDTA was added to give a final concentration of 10 mM and the cleared supernatant collected following centrifugation at 14,000 ×g for 10 min at 4 °C. Protein concentration was measured by BCA (Pierce). Immunoprecipitation (IP) was performed using 400 μg protein per reaction with either 4 μg FOXM1 antibody (sc-502) or Rabbit IgG (Cell Signaling) using either the supplied low or high IP buffer supplemented with X1 protease inhibitor cocktail and in some samples 1 mM DTT, in a final volume of 500 μL. Incubation was carried out overnight at 4 °C with end-to-end rotation. Pull-down was performed by the addition of 50 μL pre-washed protein A magnetic beads (Invitrogen) for 1 h followed by X6 washes with IP buffer. After the final wash, beads were collected by centrifugation and resuspended in 15 μL of X1 Novex sample buffer (Invitrogen) and heated at 70 °C for 10 min to release the bound proteins. Western blotting was performed as detailed above using antibodies for: FOXM1 (sc-502) 1:1,000, GFP (ab290) 1:5,000, LIN9 (ab71887), B-Myb (sc-724) 1:1,000 and TF2B (sc-225) 1:1,000, with LiCor IRDye secondary antibodies; 680LT goat anti-rabbit IgG and 800LT goat anti-mouse IgG both at 1:10,000 and 680LT donkey anti-goat IgG at 1:15,000 and visualized using an Odyssey scanner.

### Statistical analysis

All statistical analyses not described above were performed with GraphPad prism software or R [80]. The tests for difference between means were performed using the two-tailed Student’s t-test. If not otherwise stated, *P* value <0.05 were considered statistically significant. Error bars represent standard deviations.

## Additional files

Additional file 1:
**Supplementary Methods, Tables and Figures as mentioned in the text.**
Additional file 2:
**FOXM1 binding peaks.** Excel spreadsheet containing location of FOXM1 binding peaks identified using MACS in replicate ChIP-seq experiments performed with endogenous FOXM1 and WT and DBD mutated GFP-FOXM1 in HEK293 cell lines. Additional file 3:
**Differential binding data.** Excel spreadsheet with output from DBA. Differential bound peaks (FDR <0.05) were identified using edgeR. Additional file 4:
**RIME proteomics data.** Excel spreadsheet with details of proteins identified following RIME analysis of FOXM1 interacting proteins. Experiment was performed using four biological replicates analyzed in two batches shown on sheets 1 and 2. Additional file 5:
**Phosphorylation sites.** Excel spreadsheet with details of phosphorylation sites identified following RIME analysis in four replicates of WT GFP-FOXM1 or R286A DBD mutant GFP-FOXM1.

## Abbreviations

Bp: Basepair
CEAS: Cis-regulatory element annotation system
ChIP: Chromatin immunoprecipitation
co-IP: co-Immunoprecipitation
DBA: Differential binding analysis
DBD: DNA binding domain
DTT: Dithiothreitol
ER: Estrogen receptor
FDR: False discovery rate
FKH: Forkhead
GO: Gene ontology
GREAT: Genomic region enrichment analysis tool
GSEA: Gene set enrichment analysis
kb: kilobase
qPCR: Quantitative polymerase chain reaction
REViGO: Reduce and visualize gene ontology
TSS: Transcription start site
