# Supplementary material for: FOXM1 binds directly to non-consensus sequences in the human genome
Source: Genome Biol. 2015 Jun 23;16(1):130. doi: 10.1186/s13059-015-0696-z (PMC4492089; doi:10.1186/s13059-015-0696-z)
Supplement: Additional file 1: — Supplementary Methods, Tables and Figures as mentioned in the text. [file 13059_2015_696_MOESM1_ESM.docx]

**Supporting Information:**

**FOXM1 binds directly to non-consensus sequences in the human genome**

Deborah A Sanders, Michael V Gormally, Giovanni Marsico, Dario Beraldi, David Tannahill and Shankar Balasubramanian

**Table of Contents:**

**Table S1** ChIP-Seq libraries used for mapping GFP-FOXM1 and FOXM1 binding sites.

**Table S2** Enriched motifs present in endogenous and GFP-FOXM1 binding peaks

**Table S3** Enriched GO processes associated with FOXM1 binding peaks

**Table S4** Enriched motifs present in WT and DBD mutant GFP-FOXM1 binding peaks

**Table S5** Top enriched GO processes associated with FOXM1 binding peaks

**Table S6** RIME analysis of GFP-FOXM1 WT versus R286A DBD mutant

**Table S7** FOXM1 phosphorylation sites identified by RIME analysis of GFP-FOXM1 WT versus R286A DBD mutant

**Figure S1** FOXM1 WT and mutant transactivation activity in luciferase reporter assay

**Figure S2** Detection of GFP-tagged FOXM1 in HeLa Trex cells

**Figure S3** FOXM1 isoform expression in GFP-FOXM1 cell lines

**Figure S4** Sequencing traces for GFP-FOXM1 cell lines

**Figure S5** Immunofluorescence staining of 239 TetR GFP-FOXM1 expressing cells

**Figure S6** ChIP-qPCR for HEK293 cells expressing GFP or GFP-FOXM1.

**Figure S7** Binding peaks identified only in the GFP-FOXM1 dataset.

**Figure S8** ReViGO analysis of the peaks present in both FOXM1 and GFP-FOXM1

**Figure S9** Genomic binding is reduced in the GFP-FOXM1 DBD mutants compared to the WT.

**Figure S10** Venn diagrams showing overlap of proteins identified by RIME analysis.

**Figure S11** GeneGo analysis of the FOXM1 interacting proteins identified by RIME

**Figure S12** RIME LC/MS-MS spectra showing identified PTM

**Figure S13** ChIP-seq binding data for promoter regions for WT and mutant GFP-FOXM1

**Figure S14** EMSA analysis to show interaction of FOXM1 DBD with fluorescently tagged DNA.

**Supplementary References**

### Table S1. ChIP-Seq libraries used for mapping GFP-FOXM1 and FOXM1 binding sites.

| **Library ID** | **Cell line** | **ChIP** | **Replicate** | **N. reads** | **N. Aligned** |
| --- | --- | --- | --- | --- | --- |
| 065 | 293 Flp | input |  | 37457050 | 26017691 |
| 066 | 293 Flp | FOXM1 | 1 | 20701441 | 17883635 |
| 067 | 293 Flp | FOXM1 | 2 | 21215792 | 18251619 |
| 068 | 293 GFP-foxm1 WT | GFP | 1 | 22016501 | 19142136 |
| 069 | 293 GFP-foxm1 WT | GFP | 2 | 29128524 | 25184917 |
| 070 | 293 GFP-foxm1 WT | input |  | 32665697 | 28202597 |
| 071 | 293 GFP-foxm1 WT | GFP | 1 | 32854194 | 28304487 |
| 072 | 293 GFP-foxm1 H/A mut | GFP | 1 | 29622647 | 25410051 |
| 075 | 293 GFP-foxm1 WT | input |  | 36709129 | 31688863 |
| 076 | 293 GFP-foxm1 WT | GFP | 2 | 32292059 | 27791205 |
| 077 | 293 GFP-foxm1 WT | GFP | 3 | 38437736 | 33073351 |
| 078 | 293 GFP-foxm1  H/A | GFP | 2 | 38484884 | 33192468 |
| 079 | 293 GFP-foxm1  H/A | GFP | 3 | 38376213 | 32890834 |
| 080 | 293 GFP-foxm1  R/A | GFP | 1 | 43946596 | 37574604 |
| 081 | 293 GFP-foxm1  R/A | GFP | 2 | 52982326 | 45319526 |

### Table S2. Enriched motifs present in endogenous and GFP-FOXM1 binding peaks

Adjusted p-values for enrichment of transcription factor motifs in peaks called on the FOXM1 and GFP-FOXM1 ChIP-Seq. Column “GFP-FOXM1 only” refers to peaks found in GFP-FOXM1 but not in FOXM1.

| **TF** | **FOXM1** | **GFP-FOXM1** | **GFP-FOXM1_only** |
| --- | --- | --- | --- |
| NFYB | 4.44E-25 | 3.03E-177 | 5.03E-144 |
| NFYA | 1.50E-21 | 3.84E-137 | 9.79E-102 |
| HAP3 | 2.53E-20 | 6.09E-141 | 3.26E-115 |
| HAP4 | 2.27E-16 | 2.95E-93 | 9.25E-67 |
| HAP5 | 5.96E-16 | 3.16E-94 | 1.07E-75 |
| SP2 | 8.62E-12 | 1.62E-150 | 8.34E-118 |
| GATA2 | 1.20E-10 | 1.79E-17 | 7.54E-09 |
| FOXP2 | 2.25E-10 | 7.00E-146 | 1.73E-152 |
| SP1 | 7.98E-10 | 5.56E-170 | 1.64E-135 |
| EGR1 | 1.24E-09 | 3.27E-101 | 3.67E-88 |
| GATA3 | 2.76E-09 | 2.92E-13 | 2.80E-11 |
| E2F3 | 6.20E-09 | 5.60E-30 | 3.88E-31 |
| Gata1 | 9.36E-09 | 3.70E-10 | 3.18E-08 |
| FOXP1 | 1.03E-08 | 5.02E-104 | 1.29E-100 |
| slp1 | 1.43E-08 | 6.12E-79 | 8.84E-78 |
| E2F6 | 1.75E-08 | 2.63E-44 | 4.07E-33 |
| Ets1 | 2.74E-08 | 4.47E-95 | 4.05E-91 |
| GZF3 | 7.37E-08 | NA | NA |
| Gata4 | 1.05E-07 | 6.37E-08 | 0.03025 |
| EBF1 | 3.30E-07 | NA | NA |
| Foxo1 | 5.02E-07 | 1.14E-115 | 4.59E-122 |
| GAT1 | 6.22E-07 | 0.001157 | NA |
| FKH1 | 7.27E-07 | 6.43E-78 | 1.81E-73 |
| FOXA1 | 1.61E-06 | 1.69E-62 | 3.49E-59 |
| Foxa2 | 2.73E-06 | 1.08E-73 | 1.99E-73 |
| ELK4 | 2.73E-06 | 2.85E-112 | 8.02E-117 |
| HCM1 | 6.66E-06 | 2.13E-83 | 5.13E-79 |
| FOXF2 | 9.66E-06 | 1.38E-89 | 2.71E-89 |
| UGA3 | 1.11E-05 | 2.80E-17 | 5.57E-14 |
| EGR2 | 1.88E-05 | 3.69E-62 | 9.06E-56 |
| FLI1 | 2.55E-05 | 1.45E-109 | 2.98E-107 |
| FKH2 | 2.83E-05 | 3.96E-131 | 1.18E-135 |
| KLF5 | 3.12E-05 | 9.41E-125 | 7.58E-109 |
| PHA-4 | 9.59E-05 | 2.66E-60 | 1.41E-64 |
| btd | 0.0002041 | 6.64E-104 | 1.20E-96 |
| ELF1 | 0.0002229 | 4.01E-113 | 5.78E-121 |
| GABPA | 0.0003913 | 3.41E-113 | 1.15E-107 |
| hkb | 0.0005128 | 6.66E-90 | 4.20E-73 |
| CST6 | 0.002374 | 5.09E-46 | 5.81E-46 |
| Gfi1 | 0.003149 | 5.66E-16 | 6.70E-13 |
| CG4328 | 0.004638 | 3.43E-11 | 5.76E-10 |
| bZIP910 | 0.009309 | 1.40E-37 | 4.32E-39 |
| Klf4 | 0.01313 | 3.62E-96 | 7.40E-82 |
| Erg | 0.01843 | 7.09E-77 | 4.25E-80 |
| FOXO3 | 0.01956 | 3.67E-63 | 2.02E-64 |
| fkh | 0.03707 | 7.17E-38 | 2.65E-48 |
| Abd-B | NA | 4.14E-17 | 1.09E-14 |
| BLMP-1 | NA | 0.02311 | NA |
| BZR1 | NA | 1.01E-10 | 5.83E-10 |
| Bhlhe40 | NA | 0.001109 | NA |
| CAD | NA | 5.79E-26 | 1.02E-20 |
| CBF1 | NA | 2.58E-21 | 9.22E-18 |
| CDX2 | NA | 4.85E-28 | 5.95E-27 |
| CEBPB | NA | NA | 0.001357 |
| CG42234 | NA | 8.94E-05 | 0.001123 |
| CHA4 | NA | 5.09E-40 | 1.93E-35 |
| CIN5 | NA | 1.65E-12 | 2.27E-11 |
| CREB1 | NA | 2.97E-63 | 4.84E-55 |
| CRZ1 | NA | NA | 0.0007136 |
| CTCF | NA | 4.53E-212 | 1.43E-224 |
| D | NA | 0.002663 | 0.01827 |
| DAF-12 | NA | 1.42E-07 | 7.73E-12 |
| DYT1 | NA | 1.38E-16 | 8.89E-11 |
| E2F4 | NA | 6.34E-15 | 6.46E-14 |
| EHF | NA | 3.31E-19 | 3.21E-20 |
| ELF5 | NA | 5.34E-23 | 2.67E-23 |
| ELK1 | NA | 1.99E-58 | 1.68E-63 |
| EOR-1 | NA | 6.14E-14 | 4.31E-10 |
| ERF1 | NA | 4.61E-12 | 3.07E-11 |
| EWSR1-FLI1 | NA | 9.08E-18 | 5.83E-17 |
| Eip74EF | NA | 1.27E-86 | 1.21E-85 |
| EmBP-1 | NA | 6.29E-06 | 7.63E-07 |
| FEV | NA | 3.31E-65 | 8.46E-75 |
| FHL1 | NA | 4.02E-10 | 1.65E-05 |
| FOS | NA | NA | 0.003089 |
| FOSL1 | NA | NA | 0.00333 |
| FOSL2 | NA | 0.02611 | NA |
| FOXD1 | NA | 9.93E-70 | 1.72E-74 |
| FOXH1 | NA | 5.80E-15 | 1.36E-14 |
| FOXI1 | NA | 2.67E-30 | 6.49E-26 |
| Foxd3 | NA | 5.55E-26 | 3.07E-22 |
| Foxq1 | NA | 1.84E-45 | 2.37E-38 |
| GCN4 | NA | 0.0007242 | 0.0008896 |
| GCR2 | NA | NA | 2.05E-05 |
| HAC1 | NA | 3.83E-07 | 0.0002738 |
| HLF | NA | 4.35E-20 | 2.85E-18 |
| HMG-I/Y | NA | 8.65E-11 | 3.13E-06 |
| HNF1A | NA | 1.20E-05 | 0.04418 |
| HY5 | NA | 7.62E-05 | NA |
| Hoxa9 | NA | 8.56E-07 | 0.00104 |
| Hoxc9 | NA | 4.81E-15 | 6.57E-13 |
| IRF1 | NA | 3.35E-25 | 1.66E-20 |
| JUN | NA | 2.44E-52 | 5.26E-48 |
| JUNB | NA | 5.59E-05 | 1.38E-05 |
| JUND | NA | 3.92E-47 | 5.84E-44 |
| Klf1 | NA | 1.77E-58 | 7.45E-44 |
| LEU3 | NA | 0.007314 | NA |
| MEF2C | NA | NA | 0.0003499 |
| MET32 | NA | NA | 1.35E-08 |
| MYC::MAX | NA | 2.18E-10 | 0.0005392 |
| Mad | NA | NA | 0.0006108 |
| Mycn | NA | 4.74E-12 | 2.68E-07 |
| Myod1 | NA | 7.10E-06 | 0.00482 |
| Myog | NA | 7.28E-05 | 0.02914 |
| NFATC2 | NA | 1.42E-08 | 3.38E-07 |
| NFIL3 | NA | 1.54E-05 | NA |
| NHLH1 | NA | NA | 0.005195 |
| NHP6B | NA | 0.0008173 | NA |
| NRF1 | NA | 0.02666 | NA |
| PIF3 | NA | 2.57E-12 | 3.48E-10 |
| PIF4 | NA | 0.001445 | NA |
| PIF5 | NA | 6.11E-20 | 6.59E-15 |
| PIL5 | NA | 8.33E-13 | 5.43E-11 |
| PRDM1 | NA | 1.59E-05 | 0.007484 |
| Pax2 | NA | 0.0001811 | 0.0003852 |
| RDR1 | NA | 5.09E-08 | 3.40E-12 |
| RFX2 | NA | 0.003859 | 0.006675 |
| RPN4 | NA | 3.28E-08 | 2.23E-10 |
| RREB1 | NA | 0.01359 | NA |
| RSC3 | NA | NA | NA |
| RSC30 | NA | 9.72E-06 | 3.54E-05 |
| Rfx1 | NA | NA | 0.03884 |
| SIP4 | NA | 0.0002403 | NA |
| SOC1 | NA | 2.04E-24 | 5.59E-22 |
| SPIB | NA | 1.29E-05 | 0.0001437 |
| SRY | NA | 3.11E-15 | 4.56E-09 |
| STAT1 | NA | 4.40E-16 | 4.30E-18 |
| STAT2::STAT1 | NA | 1.61E-20 | 3.63E-13 |
| STAT3 | NA | 1.86E-12 | 3.75E-10 |
| STP1 | NA | 7.44E-06 | 0.0005595 |
| Sox3 | NA | 1.19E-16 | 0.001785 |
| Sox6 | NA | 2.85E-09 | 0.006221 |
| Spi1 | NA | 3.31E-35 | 2.75E-38 |
| Stat4 | NA | 2.88E-07 | 1.39E-09 |
| Stat5a::Stat5b | NA | 5.10E-06 | NA |
| TBP | NA | 2.06E-07 | 1.16E-11 |
| TGA1 | NA | 9.54E-47 | 1.10E-48 |
| TGA1A | NA | NA | 0.02111 |
| TYE7 | NA | 3.55E-32 | 1.69E-29 |
| Tcf12 | NA | 0.0003356 | NA |
| Tcf3 | NA | 2.09E-07 | 1.45E-06 |
| USF1 | NA | 1.00E-19 | 3.92E-12 |
| USF2 | NA | 3.74E-20 | 3.32E-11 |
| YAP6 | NA | NA | 0.002137 |
| YBR239C | NA | NA | NA |
| YER184C | NA | NA | 0.01258 |
| YJL103C | NA | 0.001946 | NA |
| YKL222C | NA | NA | 0.008868 |
| YRM1 | NA | 0.0002197 | 0.005743 |
| YY1 | NA | 2.59E-15 | 8.83E-19 |
| ZBTB33 | NA | 0.02612 | NA |
| ZNF263 | NA | 7.24E-38 | 4.75E-30 |
| abi4 | NA | 5.34E-14 | 1.10E-15 |
| bZIP911 | NA | 7.05E-08 | 8.12E-10 |
| br_Z1 | NA | 0.01314 | NA |
| br_Z3 | NA | 2.74E-09 | 9.77E-05 |
| br_Z4 | NA | 9.96E-11 | 3.29E-05 |
| brk | NA | 0.0006115 | 0.01609 |
| gt | NA | 1.59E-10 | 5.87E-19 |
| znf143 | NA | 1.82E-14 | 1.25E-09 |

### Table S3. Enriched GO processes associated with FOXM1 binding peaks

Top 10 enriched GO process associated with FOXM1 binding peaks in regions found only in the GFP-FOXM1 ChIP-seq dataset.

| **Term Name** | **FDR** |
| --- | --- |
| S phase | 1.45E-11 |
| signal transduction in response to DNA damage | 6.73E-11 |
| antigen processing and presentation of peptide antigen via MHC class I | 1.89E-10 |
| translational elongation | 2.79E-10 |
| viral transcription | 3.42E-10 |
| negative regulation of gene expression, epigenetic | 7.25E-10 |
| translational termination | 3.02E-09 |
| antigen processing and presentation of peptide antigen | 1.80E-08 |
| positive regulation of cell cycle arrest | 2.34E-08 |
| M/G1 transition of mitotic cell cycle | 4.27E-08 |

### Table S4. Enriched motifs present in WT and DBD mutant GFP-FOXM1 binding peaks

Adjusted p-values for enrichment of transcription factor motifs in peaks called on the GFP-FOXM1 WT and joint R286A/H287A ChIP-Seq datasets. Column “R286A/H287A” refers to peaks found in both mutant ChIP_seq datasets.

| **Transcription Factor** | **WT_FOXM1** | **R286A/H287A** |
| --- | --- | --- |
| NFYB | 8.34E-166 | 4.70E-11 |
| NFYA | 1.39E-142 | 3.54E-12 |
| HAP3 | 3.14E-138 | 4.52E-06 |
| SP1 | 2.23E-118 | NA |
| SP2 | 2.28E-107 | NA |
| HAP5 | 6.00E-104 | 1.65E-06 |
| KLF5 | 9.03E-99 | NA |
| HAP4 | 1.50E-97 | 6.08E-06 |
| FOXP2 | 3.98E-94 | NA |
| FLI1 | 5.30E-91 | 1.57E-06 |
| ELK4 | 1.15E-86 | 1.17E-09 |
| ELF1 | 6.31E-86 | 1.64E-08 |
| FKH2 | 1.56E-82 | NA |
| GABPA | 5.71E-82 | 1.06E-08 |
| Foxo1 | 1.02E-75 | NA |
| Klf4 | 1.89E-74 | NA |
| btd | 1.84E-70 | NA |
| CTCF | 2.05E-69 | NA |
| Ets1 | 3.46E-68 | 0.0002494 |
| FOXP1 | 4.26E-67 | NA |
| Eip74EF | 4.59E-64 | 6.65E-07 |
| hkb | 2.15E-58 | NA |
| EGR1 | 8.31E-57 | NA |
| FOXF2 | 3.46E-56 | NA |
| Erg | 3.69E-56 | 0.001802 |
| slp1 | 3.18E-47 | NA |
| Foxa2 | 5.26E-46 | NA |
| FEV | 1.95E-45 | 0.0005013 |
| FKH1 | 5.95E-45 | NA |
| ELK1 | 2.15E-44 | 1.42E-06 |
| FOXD1 | 8.98E-44 | NA |
| HCM1 | 1.68E-43 | NA |
| Klf1 | 1.34E-37 | NA |
| FOXO3 | 6.37E-35 | NA |
| CHA4 | 5.78E-34 | NA |
| CREB1 | 3.79E-33 | NA |
| TGA1 | 2.56E-32 | NA |
| JUND | 5.83E-32 | NA |
| CST6 | 9.20E-32 | NA |
| PHA-4 | 7.00E-31 | NA |
| FOXA1 | 2.08E-30 | NA |
| bZIP910 | 5.25E-28 | NA |
| EGR2 | 8.78E-28 | NA |
| JUN | 1.11E-27 | NA |
| Foxq1 | 4.33E-27 | NA |
| E2F6 | 3.24E-26 | NA |
| ZNF263 | 4.87E-24 | NA |
| Spi1 | 1.50E-20 | NA |
| fkh | 2.87E-20 | NA |
| E2F3 | 1.29E-19 | NA |
| Gfi1 | 2.12E-16 | NA |
| TYE7 | 3.65E-14 | NA |
| HLF | 1.57E-13 | NA |
| znf143 | 4.42E-13 | NA |
| CAD | 7.24E-13 | NA |
| EHF | 1.52E-12 | NA |
| SOC1 | 4.87E-12 | NA |
| E2F4 | 4.34E-11 | NA |
| STAT1 | 6.32E-11 | NA |
| IRF1 | 1.39E-10 | NA |
| CDX2 | 1.29E-09 | NA |
| ELF5 | 7.43E-09 | NA |
| abi4 | 1.28E-08 | NA |
| EWSR1-FLI1 | 3.02E-08 | NA |
| STAT3 | 4.89E-08 | NA |
| FOXI1 | 5.04E-08 | NA |
| TBP | 5.55E-08 | NA |
| YY1 | 2.21E-07 | NA |
| bZIP911 | 3.16E-07 | NA |
| CBF1 | 1.08E-06 | NA |
| EOR-1 | 1.09E-06 | NA |
| RDR1 | 2.01E-06 | NA |
| CG4328 | 2.57E-06 | NA |
| gt | 2.87E-06 | NA |
| UGA3 | 3.01E-06 | NA |
| CIN5 | 1.07E-05 | NA |
| ERF1 | 1.73E-05 | NA |
| Tcf3 | 3.11E-05 | NA |
| STP1 | 4.04E-05 | NA |
| SRY | 4.30E-05 | NA |
| USF1 | 4.55E-05 | NA |
| SPIB | 0.0001137 | NA |
| br_Z3 | 0.0001318 | NA |
| Hoxc9 | 0.0001425 | NA |
| SIP4 | 0.000203 | NA |
| USF2 | 0.0002041 | NA |
| Stat5a::Stat5b | 0.0002101 | NA |
| STAT2::STAT1 | 0.0003003 | NA |
| NFATC2 | 0.000613 | NA |
| NHLH1 | 0.0008508 | NA |
| br_Z4 | 0.0009904 | NA |
| Mad | 0.001027 | NA |
| FHL1 | 0.003201 | NA |
| NFIL3 | 0.003498 | NA |
| YJL103C | 0.004175 | NA |
| MEF2C | 0.005475 | NA |
| Foxd3 | 0.005558 | NA |
| PIF5 | 0.005984 | NA |
| PIL5 | 0.006547 | NA |
| FOXH1 | 0.009363 | NA |
| DAF-12 | 0.01066 | NA |
| Hoxa9 | 0.01265 | NA |
| Myod1 | 0.01269 | NA |
| DYT1 | 0.01282 | NA |
| TGA1A | 0.01877 | NA |
| Stat4 | 0.02278 | NA |
| RSC3 | 0.02718 | NA |
| RSC30 | 0.04695 | NA |
| YBR239C | 0.002759 | NA |

### Table S5. Top enriched GO processes associated with FOXM1 binding peaks

Top 10 enriched GO process associated with FOXM1 binding peaks in regions found in all three cell lines (WT, H287A & R286A) or present only in the WT FOXM1 cell line.

|  | **GO Process** | **FDR** |
| --- | --- | --- |
| Regions of H287A/R286A binding | M phase | 1.08E-22 |
|  | M phase of mitotic cell cycle | 6.65E-23 |
|  | mitosis | 1.51E-22 |
|  | organelle fission | 9.54E-22 |
|  | cell cycle phase | 1.71E-20 |
|  | mitotic cell cycle | 1.44E-20 |
|  | cell cycle | 2.88E-16 |
|  | cell cycle process | 3.41E-16 |
|  | cell division | 2.19E-15 |
| Regions of FOXM1-WT only binding | chromatin assembly or disassembly | 2.69E-17 |
|  | nucleosome organization | 2.96E-14 |
|  | negative regulation of gene expression, epigenetic | 5.05E-14 |
|  | protein-DNA complex subunit organization | 5.35E-13 |
|  | translational termination | 1.90E-12 |
|  | regulation of gene expression, epigenetic | 2.67E-11 |
|  | viral transcription | 3.61E-11 |
|  | translational elongation | 4.66E-11 |
|  | RNA 3'-end processing | 1.85E-10 |
|  | macromolecular complex disassembly | 9.03E-10 |
|  |  |  |

### Table S6. RIME analysis of GFP-FOXM1 WT versus R286A DBD mutant

GFP-FOXM1 interacting proteins identified by RIME analysis, showing the number of unique peptides identified in each replicate pull-down (with no peptides identified in the GFP only control pull-down). Peptides are grouped into joint or present in either the WT or R286A based on identification in 3 out of the 4 replicates.

|  |  | **WT** | | | | **R286A** | | | |
| --- | --- | --- | --- | --- | --- | --- | --- | --- | --- |
|  |  | **1** | **2** | **3** | **4** | **1** | **2** | **3** | **4** |
| **Present in WT and R286A** | FOXM1 | 54 | 56 | 29 | 31 | 59 | 61 | 34 | 35 |
|  | LIN54 | 13 | 10 | 4 | 2 | 3 | 5 | 4 | 11 |
|  | LIN9 | 9 | 9 | 6 | 5 | 6 | 7 | 6 | 5 |
|  | PLK1 | 8 | 5 | 3 | 1 | 4 | 7 | 4 | 7 |
|  | MYBB | 8 | 5 | 1 | 0 | 4 | 3 | 1 | 5 |
|  | UHRF1 | 4 | 4 | 3 | 1 | 1 | 2 | 1 | 0 |
|  | NLE1 | 1 | 0 | 3 | 7 | 2 | 0 | 4 | 5 |
|  | PPM1G | 3 | 3 | 1 | 2 | 2 | 3 | 2 | 5 |
|  | TCEA1 | 3 | 3 | 0 | 3 | 2 | 5 | 0 | 3 |
|  | SMC3 | 4 | 2 | 1 | 0 | 2 | 3 | 4 | 7 |
|  | PHF6 | 1 | 2 | 1 | 2 | 1 | 1 | 1 | 2 |
|  | KIF4A | 3 | 1 | 0 | 2 | 2 | 2 | 1 | 5 |
|  | LA | 2 | 1 | 1 | 1 | 2 | 1 | 0 | 2 |
|  | TNPO1 | 1 | 2 | 1 | 1 | 4 | 1 | 2 | 0 |
|  | SNW1 | 3 | 1 | 0 | 1 | 2 | 4 | 1 | 3 |
|  | RBM22 | 2 | 2 | 0 | 1 | 2 | 2 | 0 | 1 |
|  | WDR70 | 1 | 3 | 0 | 1 | 1 | 1 | 0 | 1 |
|  | NDKB | 1 | 2 | 0 | 1 | 3 | 2 | 2 | 2 |
|  | NADAP | 1 | 1 | 1 | 0 | 2 | 2 | 1 | 2 |
|  | SPSY | 1 | 1 | 0 | 1 | 2 | 1 | 1 | 3 |
|  | CAF1B | 1 | 1 | 0 | 1 | 1 | 2 | 1 | 2 |
| **Present in WT only** | WDR36 | 2 | 0 | 3 | 16 | 0 | 0 | 2 | 8 |
|  | ZMYM4 | 5 | 1 | 2 | 5 | 0 | 0 | 1 | 1 |
|  | PWP2 | 2 | 1 | 1 | 5 | 0 | 0 | 0 | 1 |
|  | K2C1B | 5 | 3 | 1 | 0 | 0 | 0 | 0 | 0 |
|  | TF2B | 2 | 2 | 1 | 2 | 0 | 0 | 1 | 0 |
|  | TPX2 | 5 | 1 | 0 | 1 | 0 | 3 | 0 | 1 |
|  | ZMYM2 | 2 | 1 | 1 | 0 | 0 | 0 | 0 | 1 |
|  | DHX40 | 1 | 1 | 1 | 0 | 2 | 0 | 1 | 0 |
|  |  |  |  |  |  |  |  |  |  |
|  |  |  |  |  |  |  |  |  |  |
|  |  |  |  |  |  |  |  |  |  |
|  |  |  |  |  |  |  |  |  |  |
|  |  |  |  |  |  |  |  |  |  |
|  |  | WT | | | | R286A | | | |
|  |  | 1 | 2 | 3 | 4 | 1 | 2 | 3 | 4 |
| **Present in R286A only** | ESTD | 0 | 0 | 2 | 1 | 3 | 2 | 3 | 2 |
|  | SART3 | 0 | 3 | 0 | 1 | 2 | 3 | 1 | 1 |
|  | CDC5L | 0 | 1 | 0 | 3 | 1 | 2 | 1 | 3 |
|  | PAF1 | 2 | 0 | 0 | 0 | 1 | 2 | 1 | 2 |
|  | DDB1 | 0 | 0 | 1 | 1 | 1 | 3 | 1 | 2 |
|  | LIN52 | 2 | 1 | 0 | 0 | 1 | 1 | 1 | 0 |
|  | P85B | 0 | 0 | 0 | 0 | 0 | 2 | 3 | 4 |
|  | RFC3 | 0 | 2 | 0 | 0 | 0 | 1 | 1 | 1 |
|  | UBP5 | 0 | 0 | 0 | 1 | 6 | 6 | 0 | 1 |
|  | RECQ1 | 0 | 1 | 0 | 1 | 2 | 2 | 0 | 3 |
|  | PSME3 | 0 | 1 | 0 | 0 | 2 | 1 | 0 | 1 |
|  | RFA2 | 0 | 1 | 0 | 2 | 2 | 2 | 0 | 3 |
|  | CHAP1 | 2 | 0 | 0 | 0 | 1 | 1 | 0 | 1 |
|  | CN166 | 0 | 0 | 0 | 1 | 1 | 3 | 0 | 1 |
|  | SUGT1 | 0 | 0 | 0 | 0 | 1 | 2 | 0 | 1 |

### Table S7. FOXM1 phosphorylation sites identified by RIME analysis of GFP-FOXM1 WT versus R286A DBD mutant

Analysis of FOXM1B peptide spectra for serine and threonine phosphorylation sites using proteome discoverer identified previously described sites of phosphorylation in both the WT and mutant proteins. The highlighted modifications represent novel phosphorylation sites, those in green were identified in > samples and those in yellow <2.

| **Phospho sites** | **WT** | | | | **R286A** | | | | **Reference** |
| --- | --- | --- | --- | --- | --- | --- | --- | --- | --- |
|  | 1 | 2 | 3 | 4 | 1 | 2 | 3 | 4 |  |
| S35 | ✕ | ✕ | ✓ | ✓ | ✕ | ✕ | ✓ | ✓ | Anders *et al* [[7](#_ENREF_7)] |
| S191 | ✕ | ✕ | ✕ | ✕ | ✓ | ✓ | ✕ | ✓ | Schimmel *et al* [[8](#_ENREF_8)] |
| S251 | ✕ | ✕ | ✕ | ✕ | ✕ | ✕ | ✕ | ✕ | Chen *et al* [[9](#_ENREF_9)] |
| S361 | ✕ | ✕ | ✕ | ✕ | ✕ | ✕ | ✕ | ✕ | Tan *et al [*[*10*](#_ENREF_10)*]* |
| S436 | ✓ | ✕ | ✓ | ✓ | ✓ | ✓ | ✕ | ✓ | Anders *et al[*[*11*](#_ENREF_11)*],* Chen *et al* [[9](#_ENREF_9)] |
| S451 | ✕ | ✕ | ✕ | ✕ | ✕ | ✓ | ✕ | ✕ |  |
| S466 | ✓ | ✕ | ✓ | ✓ | ✓ | ✓ | ✓ | ✓ | Schimmel *et al*[[8](#_ENREF_8)] |
| S474 | ✕ | ✕ | ✓ | ✕ | ✕ | ✕ | ✕ | ✓ | Anders *et al*[[11](#_ENREF_11)] |
| S486 | ✕ | ✕ | ✓ | ✓ | ✕ | ✕ | ✓ | ✓ | Christensen *et al*[[12](#_ENREF_12)] |
| S490 | ✕ | ✕ | ✕ | ✓ | ✕ | ✕ | ✕ | ✕ |  |
| S491 | ✕ | ✕ | ✓ | ✓ | ✕ | ✕ | ✓ | ✓ |  |
| S493 | ✓ | ✕ | ✓ | ✓ | ✓ | ✓ | ✓ | ✓ | Anders *et al*[[11](#_ENREF_11)*],* Schimmel *et al*[[8](#_ENREF_8)] |
| T495 | ✓ | ✕ | ✓ | ✓ | ✕ | ✕ | ✓ | ✓ | Anders *et al*[[11](#_ENREF_11)] |
| T585 | ✕ | ✕ | ✓ | ✕ | ✕ | ✕ | ✕ | ✕ | Anders *et al*[[11](#_ENREF_11)]*,* Laoukili *et al*[[13](#_ENREF_13)]*,*  Major *et al*[[14](#_ENREF_14)] |
| S594 | ✓ | ✕ | ✕ | ✕ | ✓ | ✕ | ✕ | ✕ |  |
| S595 | ✓ | ✕ | ✕ | ✕ | ✓ | ✕ | ✕ | ✕ | Schimmel *et al*[[8](#_ENREF_8)] |
| T596 | ✓ | ✕ | ✕ | ✕ | ✓ | ✓ | ✕ | ✕ | Anders *et al*[[11](#_ENREF_11)]*,* Laoukili *et al*[[13](#_ENREF_13)],  Major *et al*[[14](#_ENREF_14)]*,* Schimmel *et al*[[8](#_ENREF_8)] |
| S598 | ✕ | ✕ | ✕ | ✓ | ✕ | ✕ | ✕ | ✕ |  |
| T605 | ✓ | ✕ | ✓ | ✓ | ✓ | ✕ | ✓ | ✓ | Anders *et al*[[11](#_ENREF_11)]*,* Schimmel *et al*[[8](#_ENREF_8)] |
| S608 | ✕ | ✕ | ✓ | ✓ | ✕ | ✕ | ✓ | ✓ | Kettenbach *et al*[[15](#_ENREF_15)] |
| T612 | ✓ | ✕ | ✓ | ✓ | ✓ | ✓ | ✓ | ✓ | Anders *et al*[[11](#_ENREF_11)]*,* Schimmel *et al*[[8](#_ENREF_8)] |
| S623 | ✓ | ✕ | ✓ | ✕ | ✓ | ✕ | ✓ | ✕ | Anders *et al*[[11](#_ENREF_11)] |
| S632 | ✕ | ✕ | ✕ | ✕ | ✕ | ✕ | ✕ | ✓ |  |
| S657 | ✓ | ✕ | ✓ | ✓ | ✓ | ✓ | ✓ | ✓ | Anders *et al*[[11](#_ENREF_11)], Major *et al*[[14](#_ENREF_14)] |
| S671 | ✕ | ✕ | ✕ | ✕ | ✕ | ✕ | ✕ | ✓ |  |
| S677 | ✕ | ✕ | ✓ | ✓ | ✕ | ✕ | ✓ | ✓ | Chen *et al*[[9](#_ENREF_9)] |
| S678 | ✓ | ✕ | ✓ | ✓ | ✓ | ✓ | ✓ | ✓ | Fu *et al*[[16](#_ENREF_16)] |
| S680 | ✓ | ✕ | ✓ | ✓ | ✓ | ✓ | ✓ | ✓ |  |
| S689 | ✓ | ✕ | ✓ | ✓ | ✓ | ✓ | ✓ | ✓ | Anders *et al*[[11](#_ENREF_11)]*,* Ma *et al*[[17](#_ENREF_17)] |
| S695 | ✓ | ✕ | ✕ | ✕ | ✓ | ✓ | ✓ | ✕ |  |
| S702 | ✕ | ✕ | ✓ | ✓ | ✓ | ✓ | ✓ | ✓ | Schimmel *et al*[[8](#_ENREF_8)] |
| T704 | ✕ | ✕ | ✓ | ✕ | ✕ | ✕ | ✕ | ✕ |  |
| S715 | ✓ | ✕ | ✓ | ✓ | ✕ | ✕ | ✕ | ✕ | Fu *et al*[[16](#_ENREF_16)], Schimmel *et al*[[8](#_ENREF_8)] |
| S724 | ✕ | ✕ | ✕ | ✕ | ✕ | ✕ | ✕ | ✕ | Fu *et al*[[16](#_ENREF_16)] |

### Figure S1. FOXM1 WT and mutant transactivation activity in luciferase reporter assay

Fold induction of transactivation of (A) PLK1, (B) SV40 and (C) CYP1B1 luciferase reporters following doxycycline treatment of HeLa TRex cell transfected with either FOXM1 WT or DBD mutants Data represents triplicate experiments ±SD. (*) *P*<0.05, (**) *P*<0.01, (***) *P*<0.001.

### Figure S2. Detection of GFP-tagged FOXM1 in HeLa Trex cells

Western blots of HeLa cell lysates transfected with plasmids for GFP, GFP-FOXM1 or untransfected (Control). Blots were probed with antibodies against GFP (**A**). ChIP-qPCR (**B**) following pull-down of HeLa Trex transfected cells with GFP or GFP-FOXM1. Data representative of triplicate experiments ±SD.

**
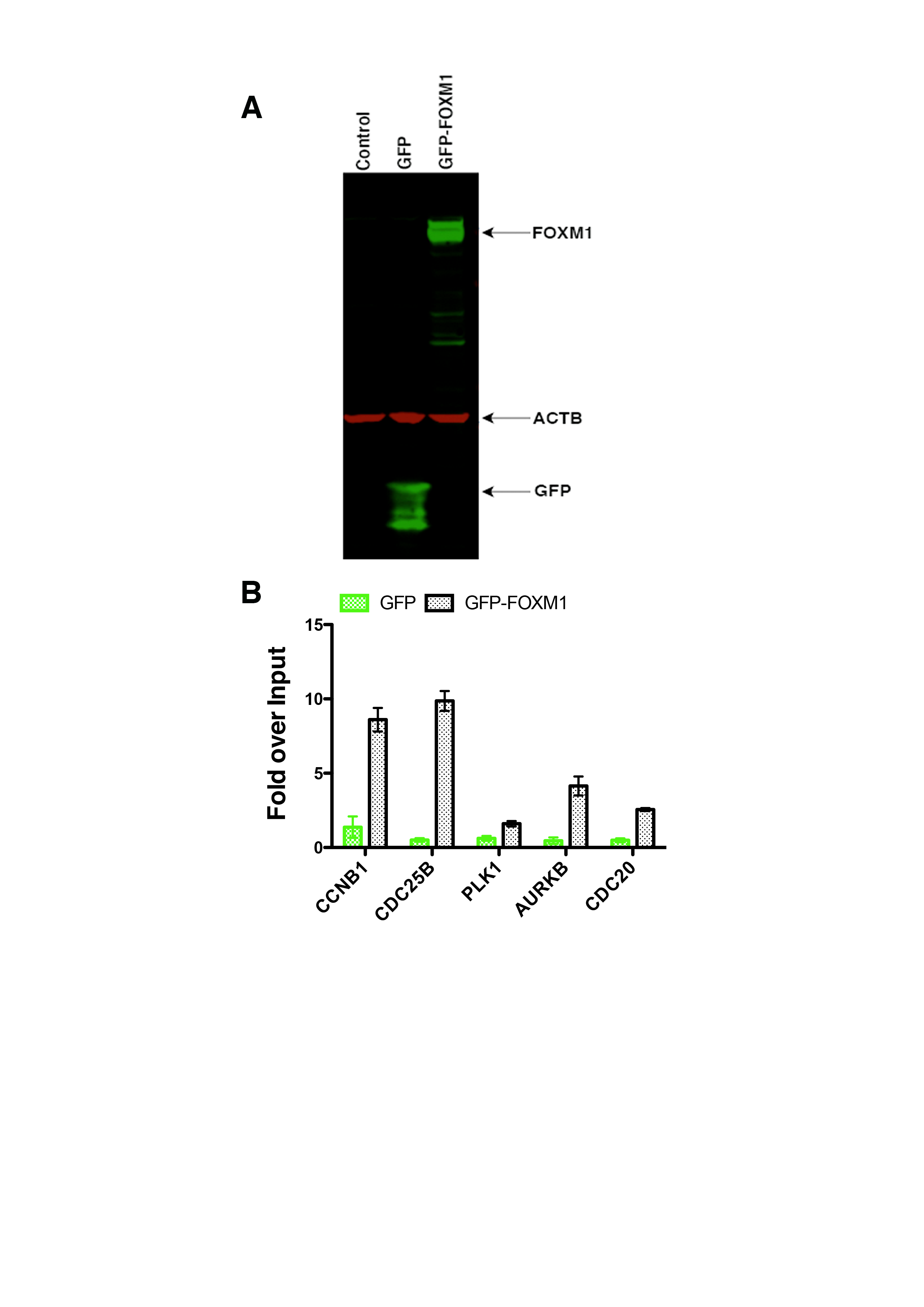
**

### Figure S3. FOXM1 isoform expression in GFP-FOXM1 cell lines

Isoform specific qPCR for FOXM1A, B & C expression in GFP-FOXM1 itreated for 24 h ± doxycycline. Data representative of triplicate experiments ±SD.


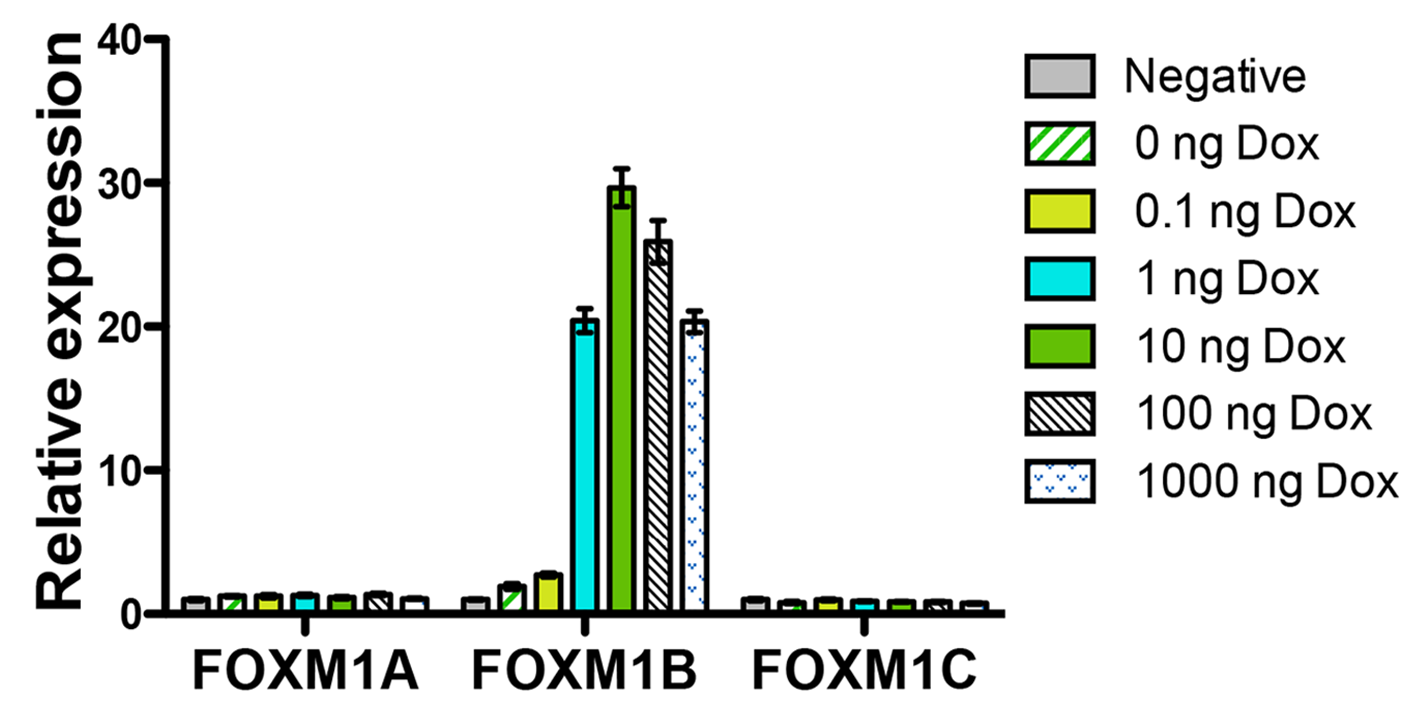


### Figure S4. Sequencing traces for GFP-FOXM1 cell lines

Sequencing traces for the WT FOXM1 and H287A, R286A mutant GFP-fusion proteins. The region of sequence shown corresponds to amino acid residues 284-290 of the FOXM1 DBD, with the mutated residues shown in red box.


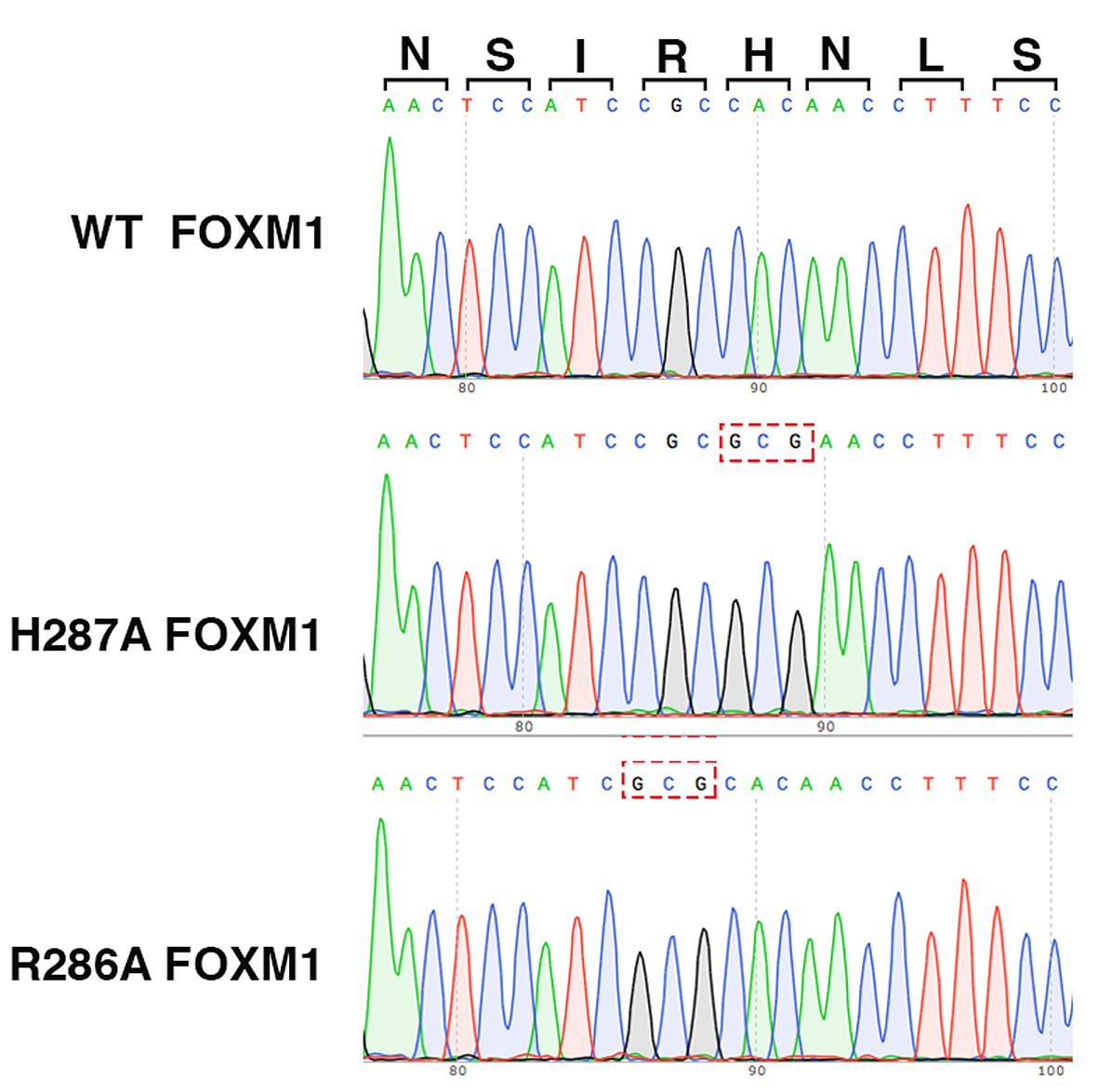


### Figure S5. Immunofluorescence staining of 239 TetR GFP-FOXM1 expressing cells

Fluorescence microscopy of HEK293 TetR GFP cell lines treated ± doxycycline (DOX) (1 μg/ml) for 24 h. Cells were stained with an anti-GFP antibody and detected using Alexa Fluor 488 and nuclei labelled with DAPI. Scale bar: 30 μM, enlarged images are X10 magnified.


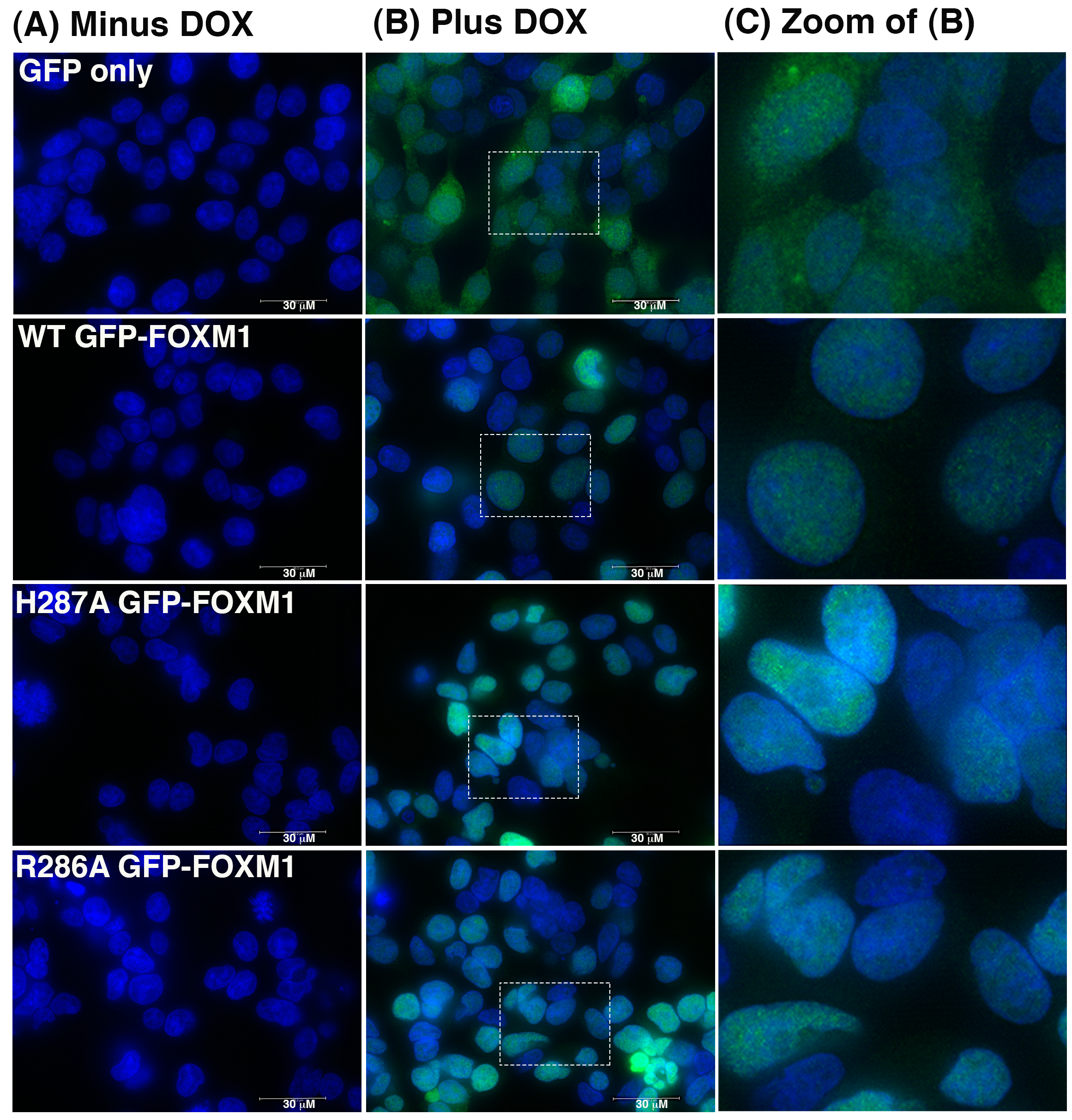


### Figure S6. ChIP-qPCR for HEK293 cells expressing GFP or GFP-FOXM1.

ChIP-qPCR results following pull-down with an anti-GFP antibody on chromatin extracted from HEK293 cells expressing either GFP-only or GFP-FOXM1 WT. Enrichment over the input control is shown at 10 known FOXM1 genomic binding sites. Data represents triplicate experiments ±SD.

### Figure S7. Binding peaks identified only in the GFP-FOXM1 dataset.

**(a)** UCSC browser images showing 10 sites with binding peaks identified by MACS in the GFP-FOXM1 ChIP-seq set but not the endogenous FOXM1 set due to low enrichment. The black bar in the upper peak trace for each region shows the GFP-FOXM1 peaks identified by MACs, whilst there was no corresponding peak for the lower trace a showing the endogenous FOXM1 pull-down.

**A**

**B**

**
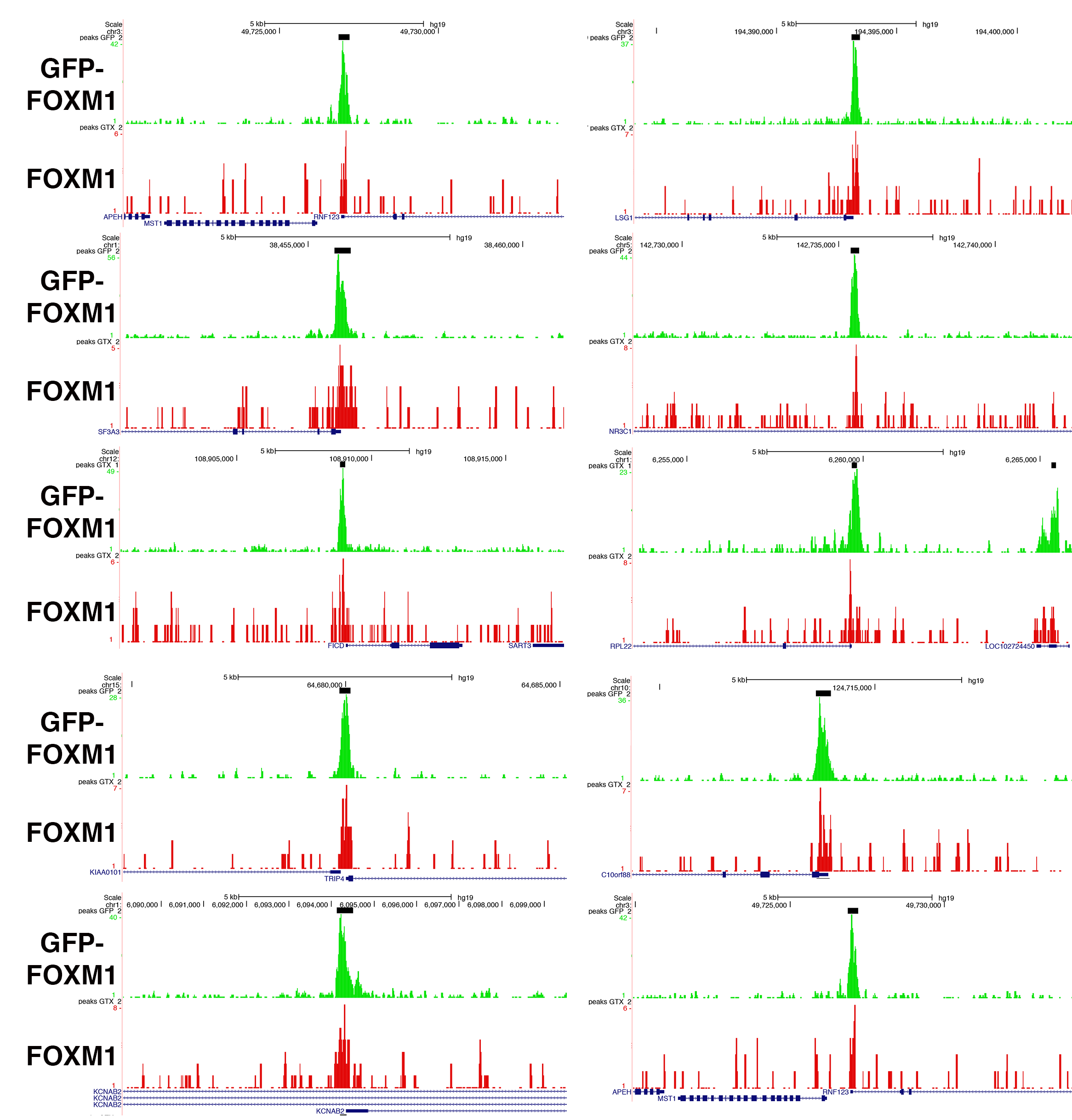
**

**(b)** Scatter plots indicating the correlation between read counts in different libraries. Coverage counts are calculated for individual libraries separately as the number of reads overlapping each of the 12,418 peak intervals identified in the foxm1_gfp data set. Pearson correlation coefficient (r) is reported at the top of each scatter plot for each pair of libraries. Results indicate that signal correlation between foxm1 and foxm1_gfp is high (0.7-0.75) even in regions where peaks are not called in the foxm1 data set suggesting that the signal detected by foxm1-GFP ChIP-Seq is not spurious. In contrast, correlation to input signal is lower (0.38-0.49). Legend for libraries: foxm1: endogenous foxm1 ChIP-Seq; foxm1_gfp: GFP-tagged foxm1 ChIP-Seq; input: input library. Replicate number indicated as _1 and _2. Color coding: black: replicate libraries for the same conditions (foxm1 and foxm1_gfp); dark red: libraries from foxm1 versus foxm1_gfp condition, all pair combinations; grey: foxm1 and foxm1_gfp versus input.


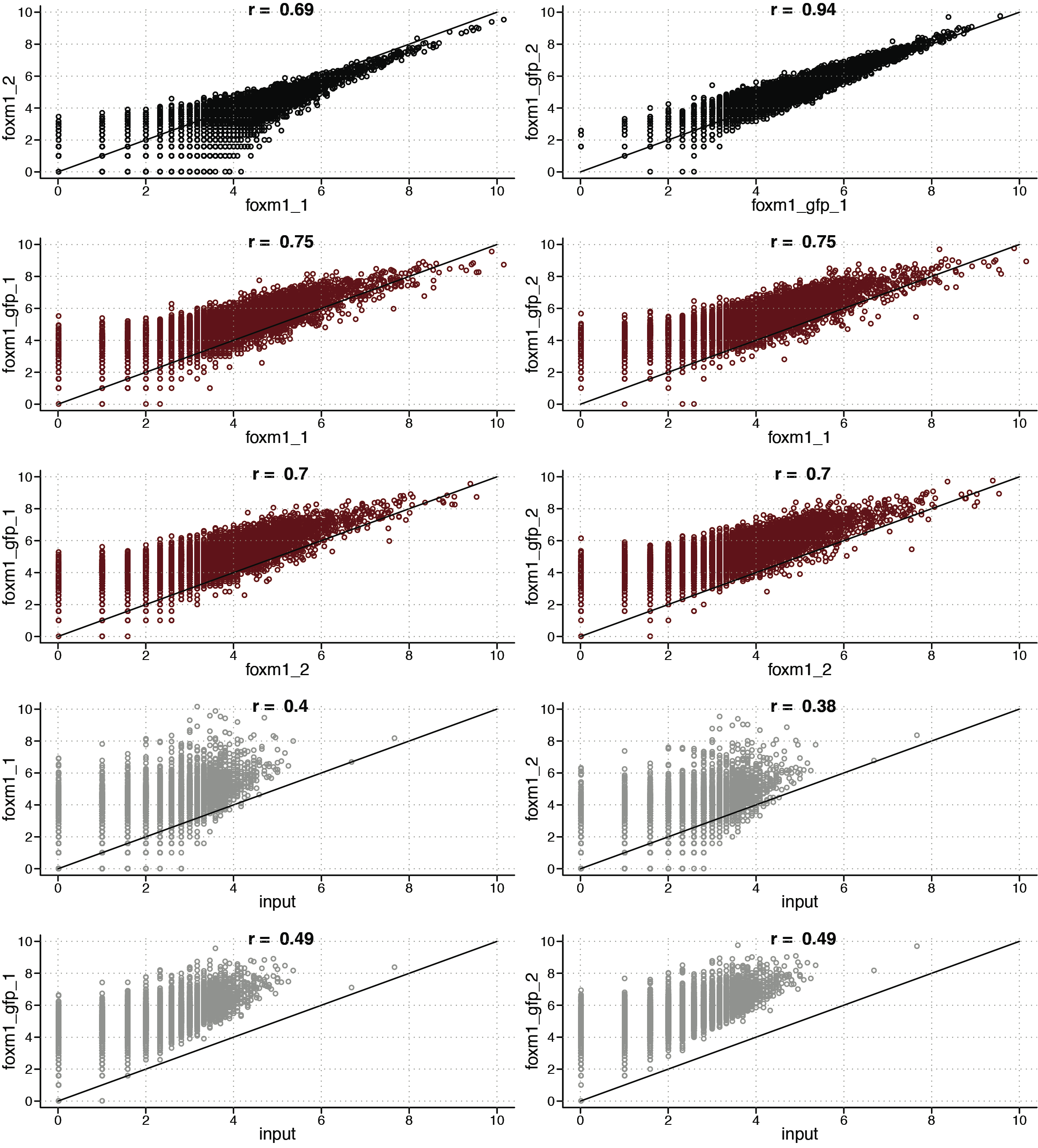


### Figure S8. ReViGO analysis of the peaks present in both FOXM1 and GFP-FOXM1

ReViGO analysis of the functional annotation of binding peaks present in both GFP-FOXM1 and endogenous FOXM1 ChIP-seq datasets. (Colour is related to p=value and the functional similarity of the GO categories is represented by the semantic space of the X&Y axes)


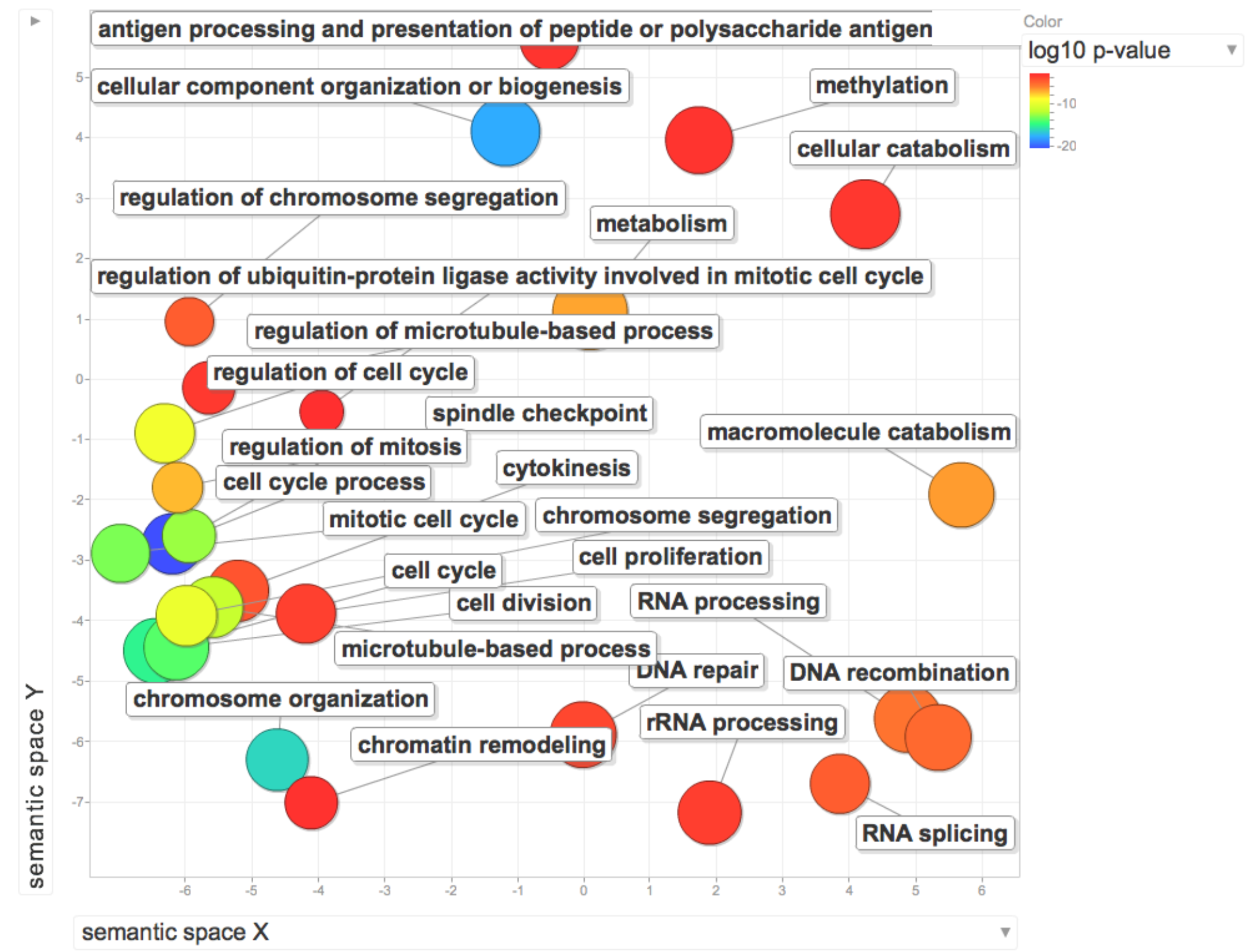


### Figure S9. Genomic binding is reduced in the GFP-FOXM1 DBD mutants compared to the WT.

ChIP-qPCR results following pull-down with an anti-GFP antibody on chromatin extracted from HEK293 celss expressing either WT or DBD mutant GFP-FOXM1. Plot shows fold enrichment over input at three known FOXM1 promoter binding sites. Data represents triplicate experiments ± SD.

### Figure S10. Venn diagrams showing overlap of proteins identified by RIME analysis.

The number of overlapping proteins identified in the RIME analysis between the WT (**A**) and R286A (**B**) replicate datasets are shown. The proteins identified in three out of the four replicates were overlapped between the WT and R286A samples (**C**).


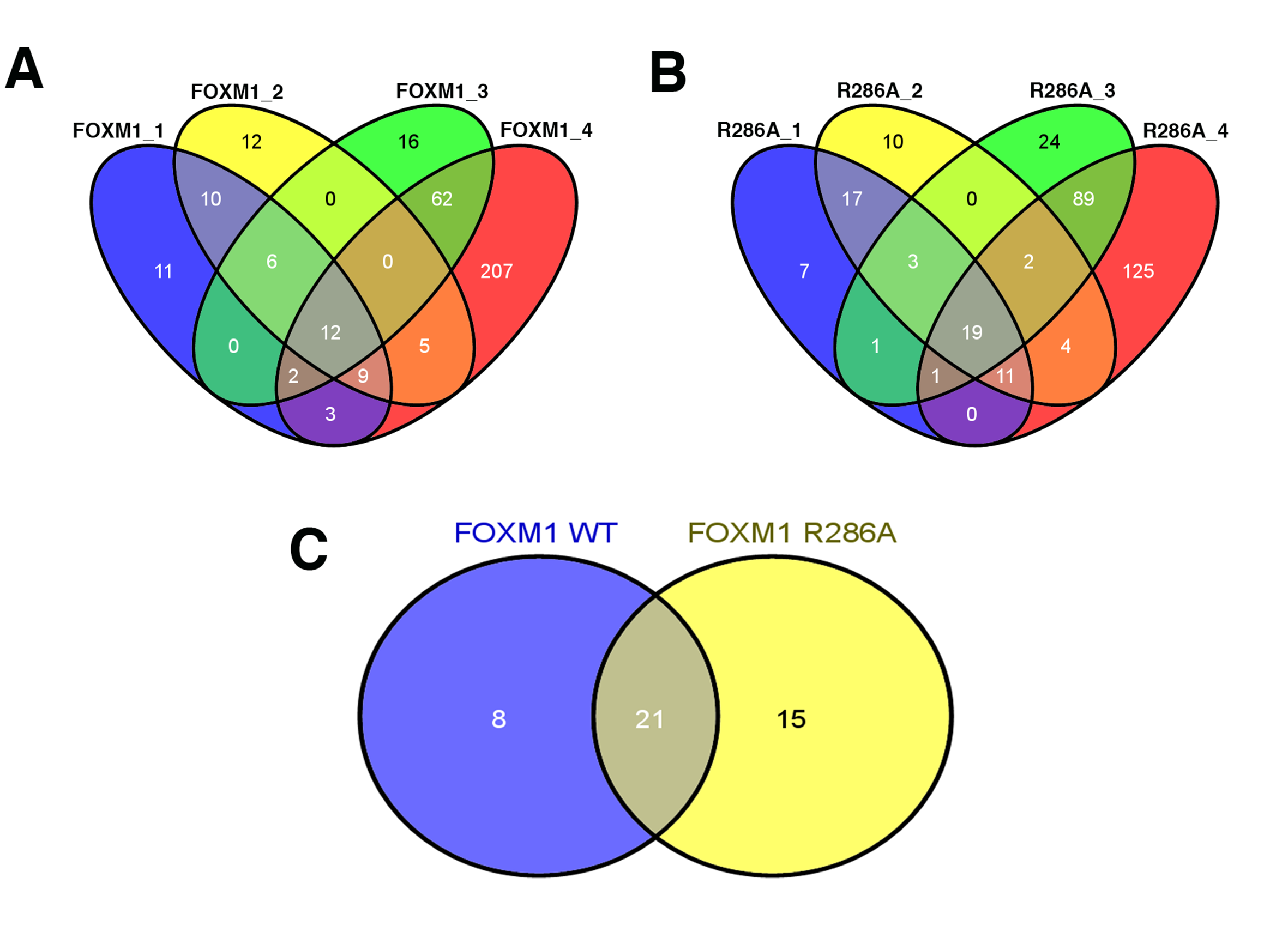


### Figure S11. GeneGo analysis of the FOXM1 interacting proteins identified by RIME

The top 10 statistically enriched biological processes are shown.


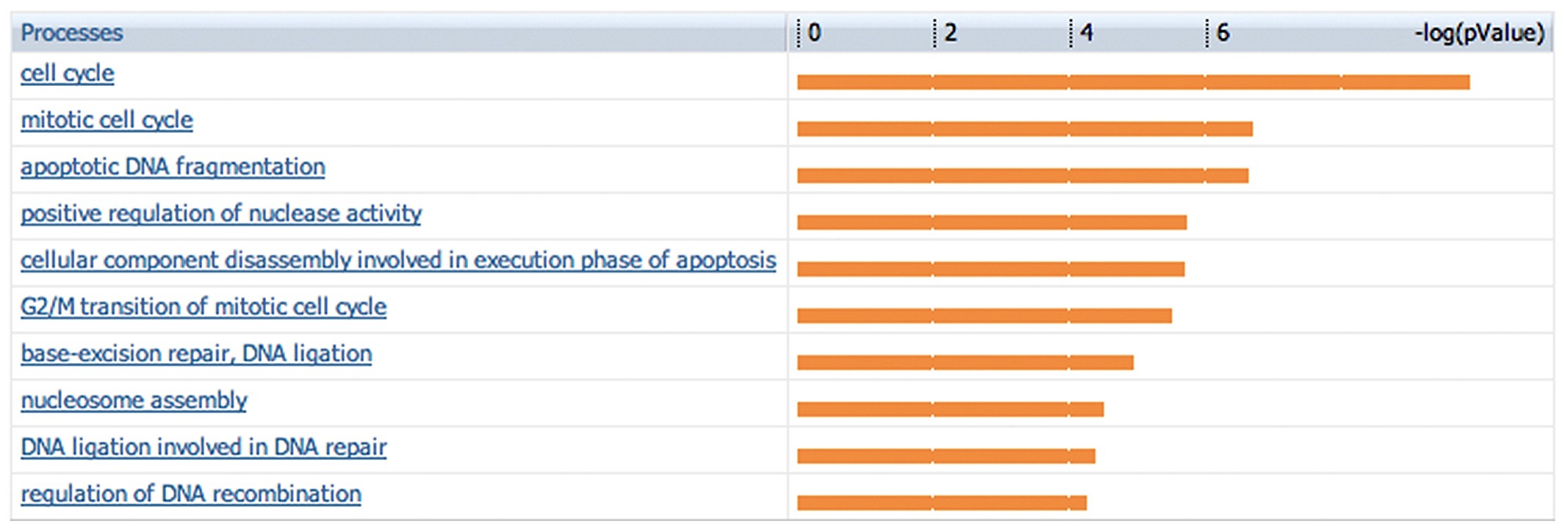


### Figure S12. RIME LC/MS-MS spectra showing identified PTM

Peptide spectra for (A) WT and (B) R286A mutant RIME samples showing position of identified phosphor serine residues.

A

**
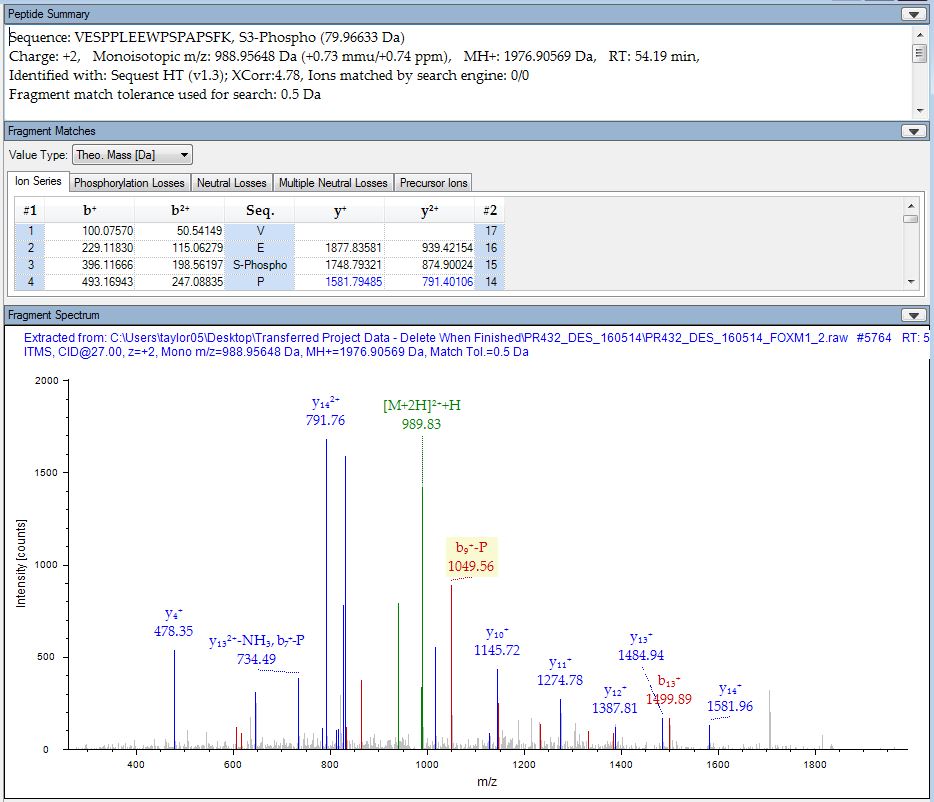
**

B

**
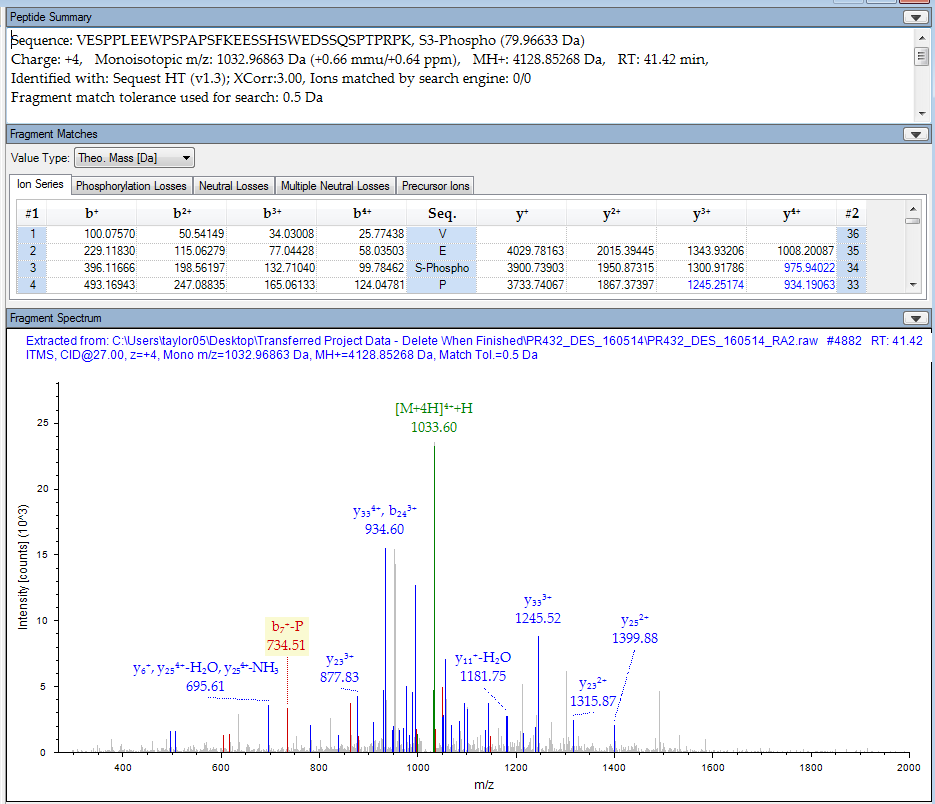
**

### Figure S13. ChIP-seq binding data for promoter regions for WT and mutant GFP-FOXM1.

UCSC browser images showing 3 promoter sites for cell-cycle regulating genes that showed differential expression levels in WT and mutant cell lines by qPCR.

**
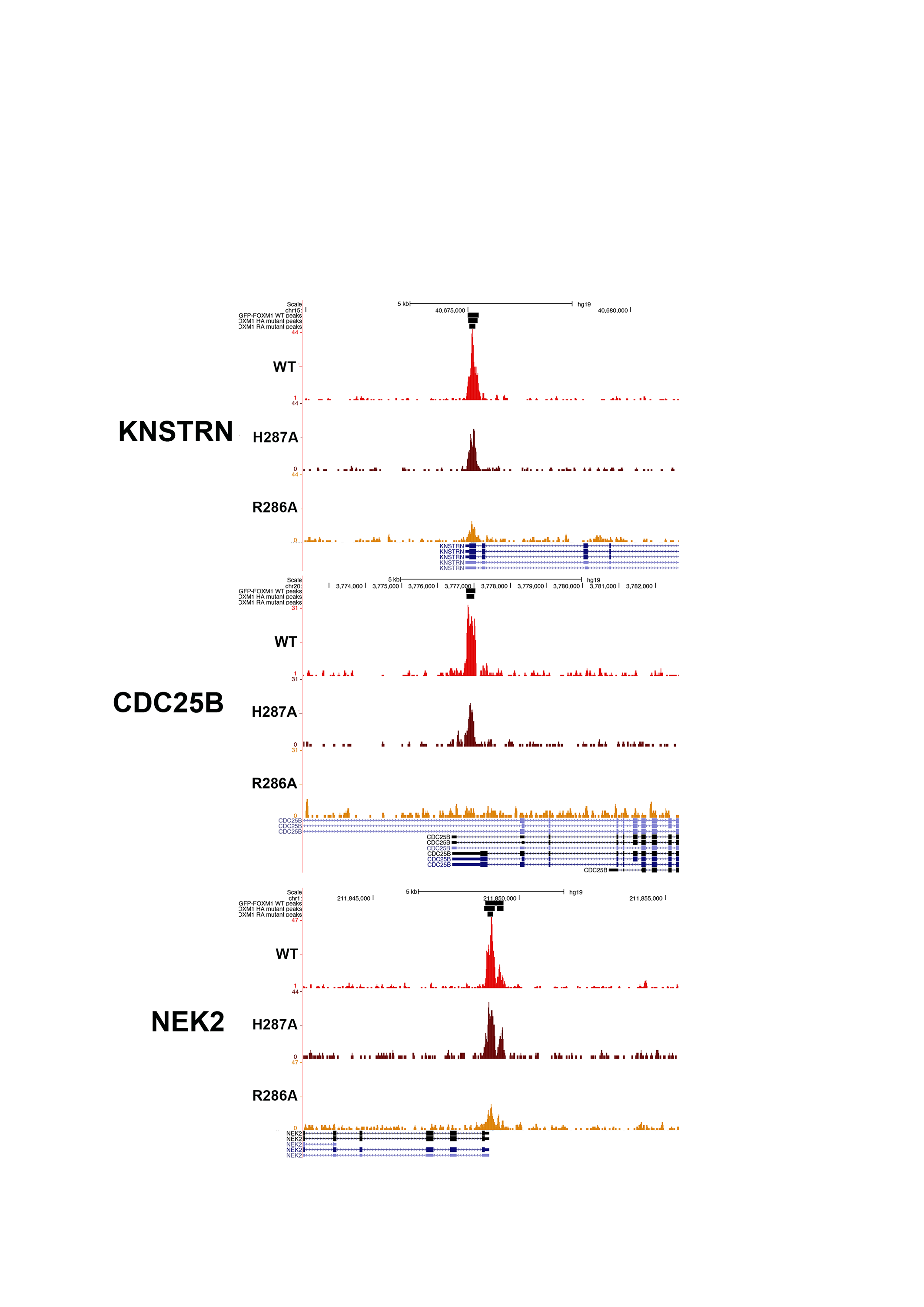
**

### Figure S14. EMSA analysis to show interaction of FOXM1 DBD with fluorescently tagged DNA.

Representative EMSA images for FOXM1 DBD showing association with fluorescently tagged dsDNA oligos (**A**) FKH consensus (**B**) from ChIP-seq binding peak in CCNB2 promoter (**C**) CCNB1 promoter (**D**) TATA box from MYC promoter and (**E**) PLK1 promoter. The K_d_ values shown for each sequence were determined from 3 experiments.

**
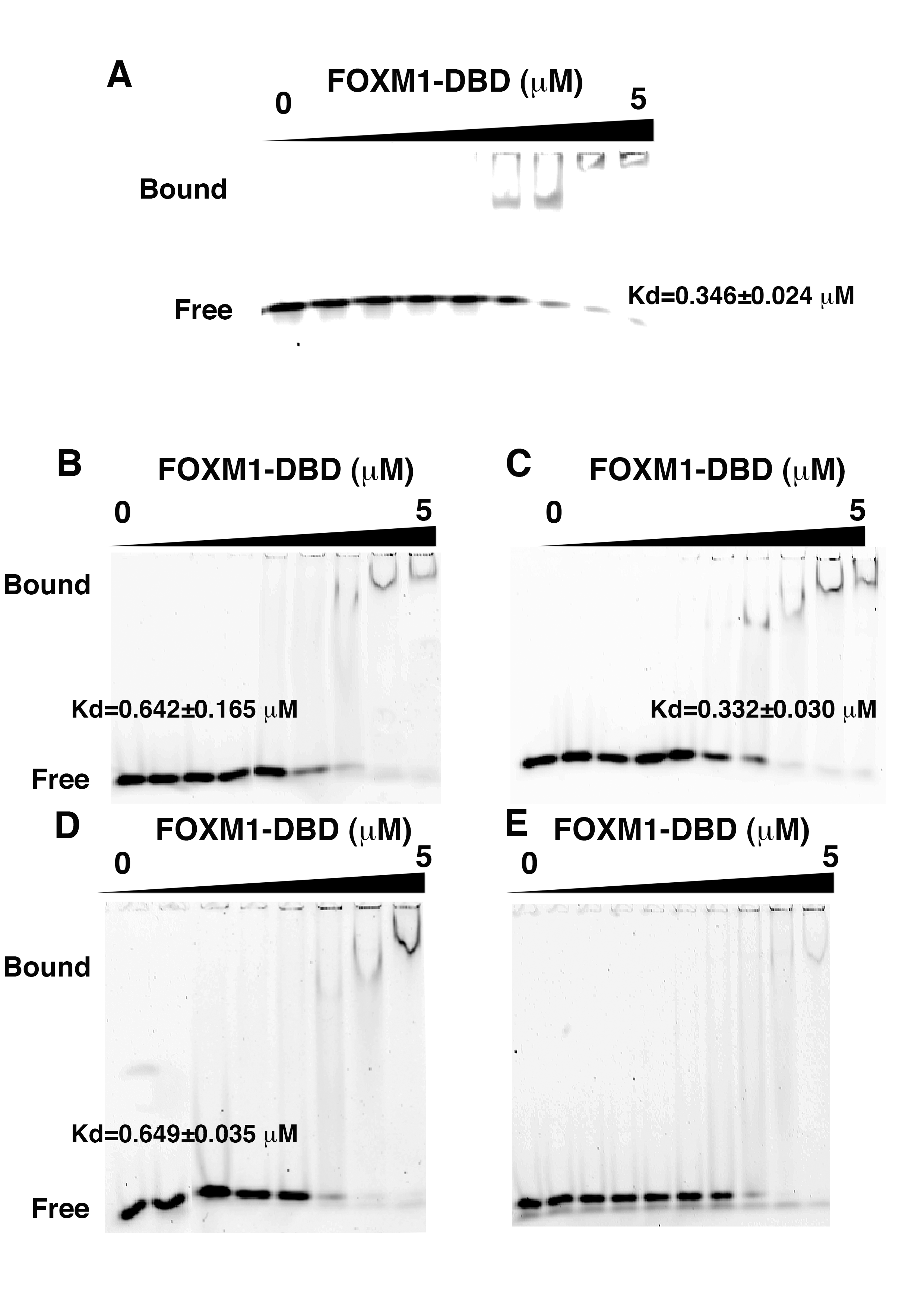
**

**Supplementary references**

1. Livak KJ, Schmittgen TD: **Analysis of relative gene expression data using real-time quantitative PCR and the 2(-Delta Delta C(T)) Method**. *Methods* 2001, 25:402-408.

2. Schmidt D, Wilson MD, Spyrou C, Brown GD, Hadfield J, Odom DT: **ChIP-seq: using high-throughput sequencing to discover protein-DNA interactions.** *Methods* 2009, 48:240-248.

3. Li H, Durbin R: **Fast and accurate short read alignment with Burrows-Wheeler transform**. *Bioinformatics* 2009, 25:1754-1760.

4. Zhang Y, Liu T, Meyer CA, Eeckhoute J, Johnson DS, Bernstein BE, Nusbaum C, Myers RM, Brown M, Li W, Liu XS: **Model-based analysis of ChIP-Seq (MACS).** *Genome biology* 2008, 9:R137.

5. Mohammed H, D'Santos C, Serandour AA, Ali HR, Brown GD, Atkins A, Rueda OM, Holmes KA, Theodorou V, Robinson JL, Zwart W, Saadi A, Ross-Innes CS, Chin SF, Menon S, Stingl J, Palmieri C, Caldas C, Carroll JS: **Endogenous purification reveals GREB1 as a key estrogen receptor regulatory factor.** *Cell Reports* 2013, 3:342-349.

6. Nesvizhskii AI, Keller A, Kolker E, Aebersold R: **A statistical model for identifying proteins by tandem mass spectrometry.** *Analytical chemistry* 2003, 75:4646-4658.

7. Abla AA, Turner JD, Sanai N: **FoxM1 is Vital in the Wnt/beta-catenin Signaling Pathogenesis of Gliomas**. *World neurosurgery* 2012, 77:594-596.

8. Schimmel J, Eifler K, Sigurethsson JO, Cuijpers SA, Hendriks IA, Verlaan-de Vries M, Kelstrup CD, Francavilla C, Medema RH, Olsen JV, Vertegaal AC: **Uncovering SUMOylation dynamics during cell-cycle progression reveals FoxM1 as a key mitotic SUMO target protein.** *Molecular cell* 2014, 53:1053-1066.

9. Chen RQ, Yang QK, Lu BW, Yi W, Cantin G, Chen YL, Fearns C, Yates JR, 3rd, Lee JD: **CDC25B mediates rapamycin-induced oncogenic responses in cancer cells.** *Cancer research* 2009, 69:2663-2668.

10. Tan Y, Raychaudhuri P, Costa RH: **Chk2 mediates stabilization of the FoxM1 transcription factor to stimulate expression of DNA repair genes**. *Molecular and cellular biology* 2007, 27:1007-1016.

11. Anders L, Ke N, Hydbring P, Choi YJ, Widlund HR, Chick JM, Zhai H, Vidal M, Gygi SP, Braun P, Sicinski P: **A systematic screen for CDK4/6 substrates links FOXM1 phosphorylation to senescence suppression in cancer cells.** *Cancer cell* 2011, 20:620-634.

12. Christensen GL, Kelstrup CD, Lyngso C, Sarwar U, Bogebo R, Sheikh SP, Gammeltoft S, Olsen JV, Hansen JL: **Quantitative phosphoproteomics dissection of seven-transmembrane receptor signaling using full and biased agonists.** *Molecular & cellular proteomics : MCP* 2010, 9:1540-1553.

13. Laoukili J, Alvarez M, Meijer LA, Stahl M, Mohammed S, Kleij L, Heck AJ, Medema RH: **Activation of FoxM1 during G2 requires cyclin A/Cdk-dependent relief of autorepression by the FoxM1 N-terminal domain.** *Molecular and cellular biology* 2008, 28:3076-3087.

14. Major ML, Lepe R, Costa RH: **Forkhead box M1B transcriptional activity requires binding of Cdk-cyclin complexes for phosphorylation-dependent recruitment of p300/CBP coactivators.** *Molecular and cellular biology* 2004, 24:2649-2661.

15. Kettenbach AN, Schweppe DK, Faherty BK, Pechenick D, Pletnev AA, Gerber SA: **Quantitative phosphoproteomics identifies substrates and functional modules of Aurora and Polo-like kinase activities in mitotic cells.** *Science signaling* 2011, 4:rs5.

16. Fu Z, Malureanu L, Huang J, Wang W, Li H, van Deursen JM, Tindall DJ, Chen J: **Plk1-dependent phosphorylation of FoxM1 regulates a transcriptional programme required for mitotic progression.** *Nature cell biology* 2008, 10:1076-1082.

17. Ma RY, Tong TH, Cheung AM, Tsang AC, Leung WY, Yao KM: **Raf/MEK/MAPK signaling stimulates the nuclear translocation and transactivating activity of FOXM1c.** *Journal of cell science* 2005, 118:795-806.
